# Small RNA and Degradome Sequencing Reveal Complex Roles of miRNAs and Their Targets in Developing Wheat Grains

**DOI:** 10.1371/journal.pone.0139658

**Published:** 2015-10-01

**Authors:** Tian Li, Lin Ma, Yuke Geng, Chenyang Hao, Xinhong Chen, Xueyong Zhang

**Affiliations:** 1 Key Laboratory of Crop Gene Resources and Germplasm Enhancement, Ministry of Agriculture / Institute of Crop Science, Chinese Academy of Agricultural Sciences, Beijing, China; 2 Shaanxi Key Laboratory of Genetic Engineering for Plant Breeding, College of Agronomy, Northwest A & F University, Yangling, Shaanxi, China; Institute of Genetics and Developmental Biology, CHINA

## Abstract

Plant microRNAs (miRNAs) have been shown to play critical roles in plant development. In this study, we employed small RNA combined with degradome sequencing to survey development-related miRNAs and their validated targets during wheat grain development. A total of 186 known miRNAs and 37 novel miRNAs were identified in four small RNA libraries. Moreover, a miRNA-like long hairpin locus was first identified to produce 21~22-nt phased siRNAs that act in *trans* to cleave target mRNAs. A comparison of the miRNAomes revealed that 55 miRNA families were differentially expressed during the grain development. Predicted and validated targets of these development-related miRNAs are involved in different cellular responses and metabolic processes including cell proliferation, auxin signaling, nutrient metabolism and gene expression. This study provides insight into the complex roles of miRNAs and their targets in regulating wheat grain development.

## Introduction

Wheat (*Triticum aestivum* L.) is the world’s major cereal crop and an important human food source. The development of wheat grain, a highly specialized organ for nutrient storage and reproductive success, is closely related to grain yield and quality [1]. The process of grain development involves meticulous and fine gene regulations at transcriptional and post-transcriptional levels [2,3]. So far, a multitude of regulatory factors that control grain development have been identified by a combination of genetic, biochemical and high-throughput sequencing approaches [1]. However, the relevant regulatory mechanism and complex regulatory network still need to be fully elucidated.

Plant small RNAs including microRNAs (miRNAs) and small interfering RNAs (siRNAs) are important regulators of gene expression at the transcriptional and post-transcriptional levels [4,5]. As well defined small RNA molecules, plant miRNAs are a class of 20–24 nt endogenous noncoding RNAs produced by highly precise excision from stem-loop precursors [6]. Plant miRNAs control the expression of genes encoding transcription factors, stress response proteins, and other important proteins, and play crucial roles in development, phytohormone signaling, nutrient homeostasis, biotic and abiotic stress adaption [5,7]. For instance, miR156 targets several different *SQUAMOSA PROMOTER-BINDING PROTEIN LIKE* (*SPL*) genes to affect many aspects of plant development, such as phase transition from the juvenile to the adult stage [8], trichome distribution during flowering [9], and plant architecture [10]. Moreover, miR396 regulates expression of *GROWTH REGULATING FACTOR* (*GRF*) genes involved in the control of cell proliferation during leaf and root development [11,12]. Therefore, accumulating evidence places miRNAs in a central position within gene expression programs that underlie plant development [13].

Increasing evidence indicates that miRNAs are also involved in the regulation of seed/grain development in crop plants. In rice, *OsSPL16/GW8*, one of miR156 target genes, controls grain size and shape by promoting cell division and grain filling [14]. Interestingly, overexpression of miR397 can improve rice yield by increasing grain size and promoting panicle branching [15]. Moreover, a high-throughput sequencing approach is widely applied to identify grain-specific miRNAs or differentially expressed miRNAs at diverse stages of grain development [16–20]. Meng *et al*. [18] identified 104 miRNAs associated with grain filling in wheat, and proposed that these miRNAs might target genes involved in various biological processes, including metabolism, transcription, cell organization and biogenesis, and signal transduction. Using similar methods, Han *et al*. [19] found that four known miRNA families and 22 novel miRNAs were preferentially expressed in developing seeds, participating in the regulation of wheat seed development and metabolism. Sun *et al*. [20] performed a whole-genome analysis of miRNAs and their targets in 11 wheat tissues, and characterized a total of 323 novel miRNAs belonging to 276 families. Nevertheless, previous studies were mostly focused on miRNA expression and novel miRNA discovery during grain development, whereas systematic experimental confirmation of the miRNA targets in the wheat grain was relatively deficient, especially for wheat-specific miRNAs [20].

Degradome sequencing is an efficient tool to identify small RNA targets on a large scale in plants [21–23]. In the present study, we aimed to identify development-associated miRNAs and their regulated targets in wheat grains at different days post anthesis (DPA) using small RNA and degradome sequencing. Four small RNA libraries (7, 14, 21 and 28 DPA grains) and two degradome libraries were sequenced. A total of 186 known miRNAs and 37 novel miRNAs were identified in our libraries. Interestingly, a miRNA-like long hairpin locus was first identified in wheat to produce 21~22-nt phased trans-acting siRNAs (ta-siRNAs). Based on sequencing data, 55 miRNA families were differentially expressed during grain development. Among these differentially expressed miRNA families, almost half of their targets were confirmed by degradome sequencing. These validated targets are involved in different cellular responses and metabolic processes, indicating the complex roles of miRNAs and their targets in the regulation of grain development.

## Materials and Methods

### Plant material and RNA isolation

Wheat (*Triticum aestivum* cv. Chinese Spring) was grown at the experimental field of Chinese Academy of Agricultural Sciences in Beijing under normal agronomic conditions. Wheat grains were collected at 7, 14, 21 and 28 DPA, snap-frozen in liquid nitrogen, and then stored at –80°C for further use. Total RNA was extracted from the frozen grains using Concert^TM^ Plant RNA Reagent (Invitrogen) according to the manufacturer’s instructions. RNA integrity was evaluated by Agilent 2100 Bioanalyzer (Agilent Technologies).

### Small RNA and degradome library construction and sequencing

To construct four small RNA libraries, total RNA (20 μg) from 7, 14, 21 and 28 DPA grains was separated by 15% polyacrylamide gel electrophoresis, and RNA molecules in the range of 18–30 nt were enriched and ligated to the 5′ and 3′ RNA adapters. The samples were used as templates for cDNA synthesis and the resulting cDNA was amplified to generate sequencing libraries. High-throughput sequencing of the four libraries was performed on an Illumina Hiseq 2000 platform.

For two degradome library construction, RNA samples were prepared as follows: equal quantities (20 μg) of total RNA isolated from 7 and 14 DPA grains were mixed together to construct the degradome library I, and equal quantities of total RNA from 21 and 28 DPA grains were mixed together to construct the degradome library II. Degradome cDNA libraries using sliced ends of polyadenylated transcripts were constructed based on a previously described method [21,24]. Degradome sequencing was performed on an Illumina GAIIx platform.

### Bioinformatic analysis of sequencing data

Raw reads from the small RNA libraries were first filtered to remove low-quality reads and other contaminants, and the resulting clean reads (18–28 nt) were used for subsequent analysis. Clean reads originating from rRNA, tRNA, snRNA, and snoRNA were analyzed by BLAST search against Sanger Rfam data [25]. Known miRNA sequences in our libraries were searched through BLAST analysis against the miRBase (version 21, http://www.mirbase.org), and sequences with less than 2 mismatches with known miRNAs in miRbase were considered known miRNAs. To evaluate the availability of wheat EST and unigene resources, all the clean reads were aligned against NCBI wheat EST database and DFCI Wheat Gene Index (ftp://occams.dfci.harvard.edu/pub/bio/tgi/data/Triticum_aestivum/). As described previously [26], potential miRNA precursors from the corresponding ESTs or unigenes were predicted using mireap (http://sourceforge.net/projects/mireap) and visualized by using the mfold web server [27]. Sequences were deemed as candidate novel miRNAs if they could be mapped to predicted hairpin structural precursors and were not annotated to known miRNAs or other non-coding RNAs.

Degradome analysis and identification of the sliced miRNA targets were performed according to the CleaveLand 3.0 pipeline [28]. Small RNA target prediction was run against wheat ESTs and unigenes using TargetFinder [29]. Degradome reads were mapped to wheat ESTs and unigenes to generate a degradome density file. Both the degradome density file and target predictions were compared and significant hits were identified as target plots (t-plots) [22].

The wheat small RNA and degradome sequencing data were submitted to NCBI Gene Expression Omnibus (GEO, http://www.ncbi.nlm.nih.gov/geo/) under the accession number GSE65799.

### Expression of miRNAs based on sequencing data

The abundances of known and novel miRNAs in each library were normalized as transcripts per million (TPM) as described [18]. For discovery of differentially expressed miRNAs among four libraries, the miRNA families with abundances greater than 10 TPM in at least one library were subjected to evaluate the statistical significance of the differences between the highest and lowest abundance levels of each miRNA according to the formula described [30]. miRNAs with statistically significant differences in expression were considered grain development-related miRNAs, and selected to perform hierarchical cluster analysis as described [31].

### RNA ligase-mediated 5′ RACE

To map and validate the cleavage sites in target mRNAs, RNA ligase-mediated rapid amplification of 5′ cDNA ends (5′ RACE) was performed by using the FirstChoice RLM-RACE Kit (Ambion) as described previously [32]. In brief, poly(A)^+^ mRNA was enriched from equally mixed total grain RNA using the Oligotex mRNA Mini Kit (Qiagen), and then ligated directly to the 5′ RACE adapter according to the manufacturer’s instructions. Two gene-specific primers were used for each RACE (S1 Table).

### Quantitative real-time PCR (qRT-PCR)

Quantification of miRNA expression by qRT-PCR was performed as described [33]. Briefly, 3 μg of total RNA, including miRNAs, was polyadenylated and reverse-transcribed with a poly(T) adapter into cDNAs for real-time PCR using a miRcute miRNA cDNA synthesis kit (Tiangen, China). The miRNA-specific forward primers were designed according to the entire miRNA sequences (S1 Table), and the sequence complementary to the poly(T) adapter was used as the reverse primer. For qRT-PCR analysis of target mRNAs, 3 μg of total RNA were reverse-transcribed using the SuperScript II System (Invitrogen) to generate cDNA. The qPCR assay of miRNAs and target mRNAs was performed on a 7500 Real-time PCR system (Applied Biosystems) using SYBR Premix Ex Taq (Takara). The wheat *actin* gene [AB181991] was used as the internal reference [34]. Gene expression levels were presented as fold-change calculated using the comparative C_T_ method [35]. All the primers used are listed in S1 Table.

## Results

### Overview of high-throughput sequencing data

To investigate wheat grain development, we collected grains at 7, 14, 21 and 28 DPA, which covered the most significant changes in weight and size during grain development (data not shown). Four small RNA libraries were then constructed using total RNA from 7, 14, 21 and 28 DPA grains. After high-throughput sequencing, a total of 72,851,249 clean reads (18–28 nt) representing 9,394,582 unique reads were obtained from four small RNA libraries (Table 1). Furthermore, two degradome libraries were also constructed and sequenced (Table 1). In total, 12,405,890 redundant reads were yielded from degradome library I (a mixed library of 7 and 14 DPA grains), and 10,963,967 redundant reads were generated from degradome library II (a mixed library of 21 and 28 DPA grains), respectively. By BLAST analysis, all the clean reads were aligned to the wheat ESTs and unigenes, generating 25,137,627 (34.50%) and 10,980,206 (46.98%) EST-matched reads for small RNA libraries and degradome libraries, respectively (Table 1).

**Table 1:** Read statistics in four small RNA libraries and two degradome libraries.

	Libraries	Redundant reads		Unique reads	
		Total clean	Wheat EST-matched^a^	Total clean	Wheat EST-matched^a^
Small RNA sequencing	7 DPA grains	17,133,120	5,378,231 (31.39%)	2,706,941	549,509 (20.3%)
	14 DPA grains	14,988,782	5,328,512 (35.55%)	3,367,889	663,371 (19.4%)
	21 DPA grains	30,638,998	10,306,959 (33.64%)	2,429,108	558,695 (23.0%)
	28 DPA grains	10,090,349	4,123,925 (40.87%)	890,644	240,652 (27.02%)
Degradome sequencing	Degradome library I^b^	12,405,890	5,676,935 (45.76%)	2,492,157	810,449 (32.52%)
	Degradome library II^c^	10,963,967	5,303,271 (48.37%)	2,096,312	753,205 (35.93%)

^a^ Reads matching NCBI wheat EST database and DFCI Wheat Gene Index.

^b^ A mixed library composed of 7 and 14 DPA grains.

^c^ A mixed library composed of 21 and 28 DPA grains.

The small RNA sequences can be clustered into several RNA classes such as known miRNAs, rRNA, tRNA, snRNA/snoRNA and others (S2 Table). For size distribution of small RNAs, 20–24 nt sequences accounte for more than 70% of the total clean reads in four small RNA libraries (S1 Fig). Consistent with previous findings in wheat grains [18,19], 24 nt reads are the most abundant class, approximately 35%–50% of redundant reads and 42%–60% of unique reads.

### Known miRNAs expressed in developing wheat grains

Currently, miRBase (release 21) lists 119 mature miRNA sequences cloned or predicted in wheat, far less than those in *Arabidopsis* and rice. To maximize the number of known miRNAs in our libraries, the clean reads were aligned against both miRNA sequences deposited in miRBase and other reported wheat miRNAs [18,20,36,37]. The criteria of the BLAST search required no more than two mismatches with the annotated miRNA sequences. In total, 186 known miRNAs were identified in the four small RNA libraries (S3 and S4 Tables). In addition to 102 known wheat miRNAs in miRBase and 39 reported wheat miRNAs, 45 known miRNAs were also identified based on their conservation in other plant species. For these 45 conserved miRNAs, 15 miRNA precursors could be identified based on the NCBI EST sequences, as shown in S2 Fig. Interestingly, miR395b-d were arranged in clusters (S2 Fig), indicating that the cluster arrangement of miR395 family is also conserved among monocots and dicots [38].

To accurately compare miRNA expression among the four libraries, the abundances of known miRNAs were normalized to transcripts per million (TPM). The most abundant miRNA family in our libraries was miR165/166 (5,697 TPM on the average), accounting for 37.6% of the total sequence reads from all known miRNAs. *Tritucum*-specific miR2009 [36] was the second most abundant miRNA family in the libraries with an average abundance of 4,439 TPM, suggesting that non-conserved miRNAs may constitute a large unique category of miRNAs in wheat grains. Furthermore, most identified miRNA*s had far less abundance than the corresponding miRNAs. However, several miRNA*s showed an even higher sequencing frequency than did the corresponding miRNAs, including miR169b-3p, miR9655-5p and miR9670-5p (S3 Table). On the other hand, many variants were observed with no more than 2 nt mismatches with the annotated miRNA sequences. These variants might be generated from the same loci as reported miRNAs, or by different loci in the complex wheat genome. Eleven miRNA variants were more abundant and substituted for the reported miRNA sequences such as miR156a, miR399 and miR408 (S3 and S4 Tables).

### Novel candidate miRNAs identified in the four small RNA libraries

Based on known miRNA identification, seven newly identified miRNAs were considered as new members of known wheat miRNAs, including miR2009b.2 and c.2, miR5048.2, miR5175b and c, miR9654c and miR9655b (Table 2). These seven miRNAs originate from known wheat miRNA loci at different positions or new loci with sequence homology to known wheat miRNAs. According to secondary structure prediction and criteria for annotation of plant miRNAs [6], 30 novel candidate miRNA loci were identified and named miRn1 to miRn21 (Table 2), and their stem-loop structures are shown in S2 Fig. None of these novel miRNAs have been found in other plant species including *Arabidopsis* or rice, indicating that they are wheat-specific. Compared with highly conserved miRNAs, most novel miRNAs had abundances of less than 100 TPM and might be expressed at moderate to low levels. The majority of newly identified miRNAs are 20–21 nt in length (Table 2), matching with the size of canonical miRNAs processed by Dicer-like proteins. Furthermore, almost one-half of them have corresponding sequenced miRNA*s, increasing the confidence that they are authentic wheat miRNAs.

**Table 2:** Newly identified 37 miRNAs in four small RNA libraries.

miRNA ID	Len	Sequence (5′–3′)	TPM^‡^				miR*^║^	Precursor
	(nt)		7 DPA	14 DPA	21 DPA	28 DPA		(EST No.)
tae-miR2009b.2	22	UGAGAAGGCAGAUCAUAAUAGC	67.53	57.24	32.02	73.24	Y	DR736484
tae-miR2009c.2	22	UGAGAAGGCAGAUCAUAAUAGC	67.53	57.24	32.02	73.24	Y	CK193889
tae-miR5048.2	22	UAUAUUUGCAGGUUUUAGGUCU	138.74	309.97	104.25	243.10	N	CJ629474
tae-miR5175b	21	UUCCAAAUUACUCGUCGUGAU	3.09	13.54	1.76	1.78	N	HX015772
tae-miR5175c	21	UUCCGAUUUACUCGUCGUGGU	0.35	0.20	0.20	0.00	N	HX013251
tae-miR9654c	22	UUCCGAAAGGCUUGAAGCAAAU	2.45	4.4	0.03	0.4	N	CA737852
tae-miR9655b	22	UGGCUACUUCCUUUCCCUUGCC	72.08	65.12	49.38	49.25	N	CV779317
Ta-miRn1	21	UUUCUUCUUCCUCUUCCUACC	31.69	8.14	2.38	0.30	N	CD935885
Ta-miRn2	21	UUGAGUCAUCUAUUUUGGAAC	7.53	5.54	3.85	4.06	Y	DR739812
Ta-miRn3a	21	UGGACGAGUAAUUCCGAACGG	9.28	9.81	8.88	2.97	N	CD881394
Ta-miRn3b	21	UAGACGAGUAAUUCCGAACGG	2.45	2.34	0.88	0.00	N	BJ249702
Ta-miRn4a	21	UCUGUAAACUAAUAUAAGAGC	6.71	8.27	2.02	0.00	Y	CJ706003
Ta-miRn4b	21	UCUGUAAACUAAUAUAAGAGC	6.71	8.27	2.02	0.00	Y	CJ788080
Ta-miRn5	21	UCACAAAUAUAAGAUGUUCUG	4.96	4.47	3.69	3.07	N	JV876054
Ta-miRn6a	21	UCUUCUGUAGAAAUAGGCACC	7.30	43.70	36.49	14.17	N	HX200139
Ta-miRn6b	21	UCUCCUGUAGAAAUAGGCACC	24.11	106.55	82.80	31.91	N	CA644455
Ta-miRn7a	21	UUAGAGAUUUCAAUAUGGACU	23.58	41.10	50.49	34.69	N	BJ268299
Ta-miRn7b	21	UUAGAGAUUUCAAUACGGACU	7.88	15.48	21.80	19.03	N	BJ273584
Ta-miRn7c	21	UUAGAGAUUCCACUACGGACU	4.55	8.87	6.89	6.05	N	HX036959
Ta-miRn8a	21	CUCCGUUCCAAAAUAGAUGAC	115.74	60.71	14.75	5.15	Y	HX035580
Ta-miRn8b	21	CUCCGUUCCAAAAUAGAUGAC	115.74	60.71	14.75	5.15	Y	HX163183
Ta-miRn8c	21	CUCCGUUCCAAAAUAGAUGAC	115.74	60.71	14.75	5.15	Y	HX032309
Ta-miRn9	21	CAAGUUAUGCAGUUGCUGCCU	9.51	0.47	0.00	0.00	N	HP621130
Ta-miRn10	21	CCACGACGAGUAAAUCGGAAC	3.09	2.80	1.21	0.00	N	CJ849101
Ta-miRn11	24	ACGGACUCCCCGCAGCCUCCACCC	20.08	21.82	8.68	16.95	N	GH731699
Ta-miRn12	21	AUCCAUAUUAGUUGUCGCUGA	5.19	4.80	1.37	1.49	N	CA632670
Ta-miRn13a	21	UUUAGAGAUUUCAAAUGGACU	17.74	55.37	207.45	183.34	Y	CD887655
Ta-miRn13b	21	UUUAGAGAUUUCAAAUGGACU	17.74	55.37	207.45	183.34	Y	CV782135
Ta-miRn14	21	UUCGGAAUUACUUGUCGCAGA	8.46	7.81	12.66	15.16	Y	CJ610100
Ta-miRn15	21	AUUACUUGUCUUGGAUUUGUC	20.02	10.87	19.81	0.10	Y	CJ786514
Ta-miRn16	21	AUUACUCGUCGCAGAAAUGGA	8.58	3.74	8.09	8.32	N	CD907788
Ta-miRn17	21	CAUUUCCGAGACAAGUAAUUC	52.35	27.89	8.52	0.00	Y	CK193033
Ta-miRn18	21	UCCGUCCCAUAAUAUAAGAGC	4.26	2.74	0.72	1.09	Y	CJ631979
Ta-miRn19a	20	UUUAUCACCGCCUCUUUCUC	21.83	8.61	3.26	0.40	Y	GAEF01081951
Ta-miRn19b	20	UUUAUCACCGCCUCUUUCUC	21.83	8.61	3.26	0.40	Y	GAEF01081952
Ta-miRn20	21	UCGAGAUCCAACGGCUGAGGU	8.29	6.14	2.58	5.85	N	JW008285
Ta-miRn21	21	UGUCUCGGAUAUGGAUGUAUC	2.86	2.94	9.69	20.91	Y	JV914483

‡ TPM: transcripts per million. miRNA abundance was scored according to the reads of defined miRNAs and their ± 2 nt variants on the precursors.

║ Y: miRNA* species (or ± 1 nt variants) for their corresponding miRNAs were sequenced in our small RNA libraries. N: miRNA* unsequenced.

**Fig 1:**
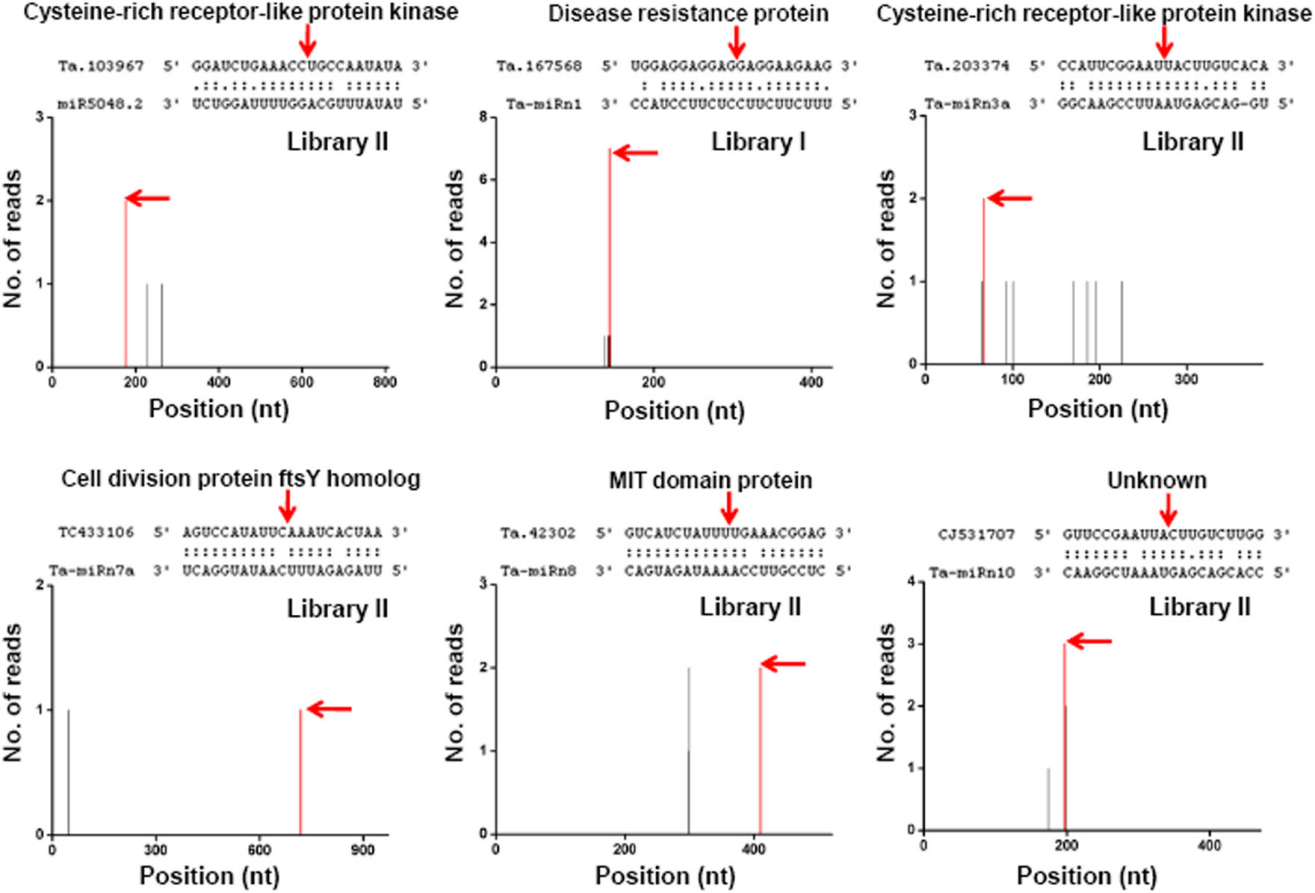
Validated targets of newly identified miRNAs using degradome sequencing. The target plots (t-plots) show sequence abundances (read counts) throughout the length of the indicated transcripts. The arrows in the t-plot indicate significant sequences consistent with miRNA-directed cleavage. miRNA:mRNA alignments along with the cleavage positions are shown above. Representative data origins from degradome libraries I or II are also indicated in the t-plots.

To further investigate the functions of new miRNAs, we predicted their targets in wheat (S5 Table). Unlike the conserved miRNAs whose targets are mainly transcription factors [7], the majority of new miRNAs are predicted to have a diverse set of target genes encoding protein kinases, disease resistance proteins, other enzymes or even unknown proteins, indicating that these miRNAs are widely involved in the regulation of various biological processes including cell metabolism and signaling transduction. Cleavage of target mRNAs mediated by some new miRNAs has been validated by degradome sequencing data (Fig 1). These verified targets had at least one degradome tag with a 5′ end precisely opposite the 10th nucleotide of the miRNAs, which is a characteristic feature of miRNA-guided slicing [22]. Both miR5048.2 and Ta-miRn3 target a class of mRNAs encoding cysteine-rich receptor-like protein kinases (Fig 1). Furthermore, Ta-miRn1, Ta-miRn7, Ta-miRn8 and Ta-miRn10 target mRNAs encoding disease resistance proteins, cell division protein ftsY homologs, a MIT (microtubule interacting and transport) domain protein and an unknown protein, respectively (Fig 1).

### Identification of a long miRNA-like hairpin locus generating phased trans-acting siRNAs

**Fig 2:**
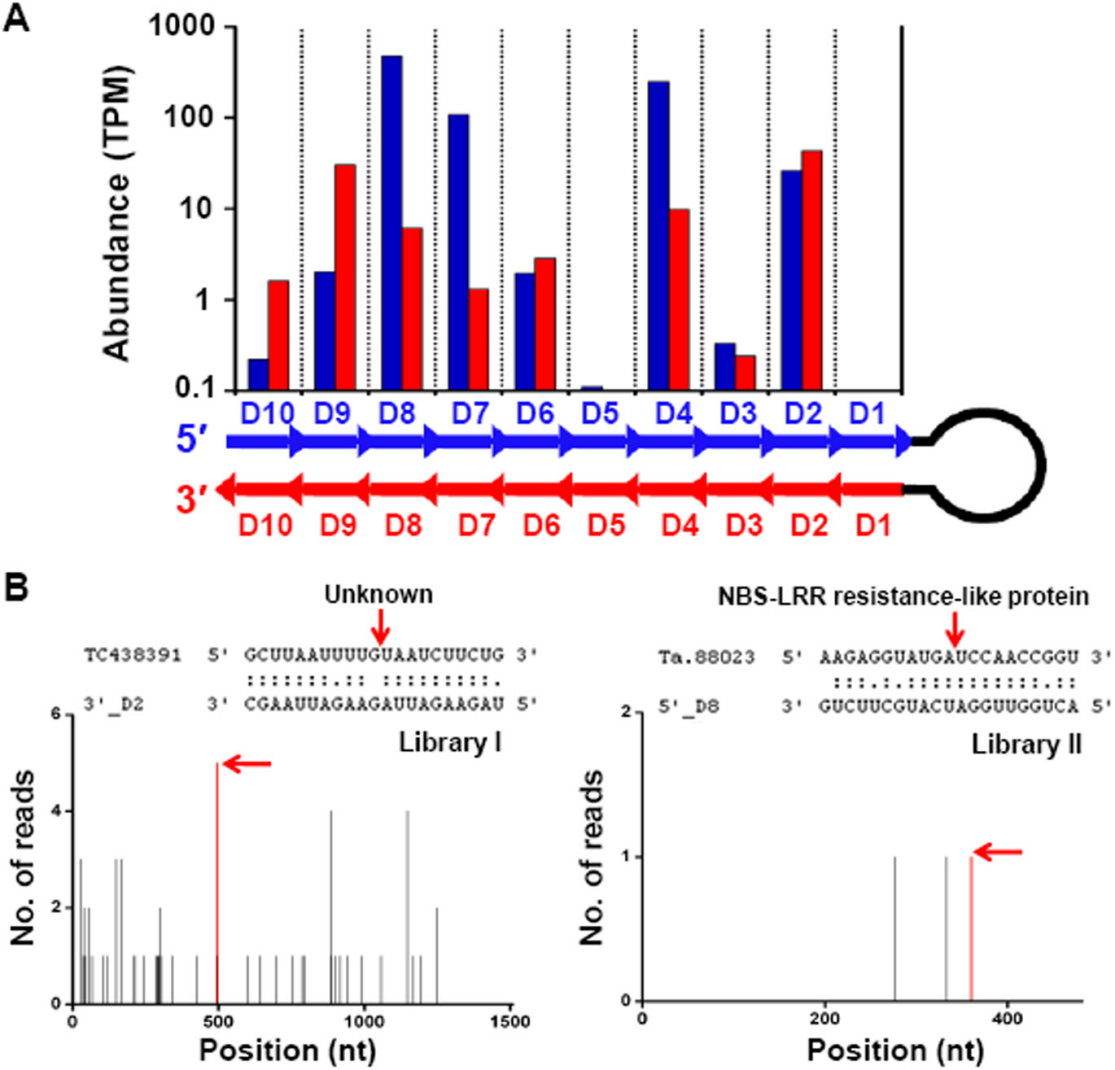
A miRNA-like long hairpin producing phased 21~22-nt ta-siRNAs. (A) Abundant small RNAs were generated from ten phases of the long hairpin, with red and blue bars representing sequences from the 5′ and 3′ arms, respectively. Their corresponding abundances are shown above as columns in the same color. (B) Degradome analysis confirmed the cleavage of target mRNAs guided by 3′_D2 and 5′_D8. The t-plots showed absolute sequence abundances over the target mRNAs, with red arrows indicating sequence reads consistent with siRNA-directed cleavage. Representative data origins from degradome libraries I or II are also indicated in the t-plots.

Trans-acting siRNAs (ta-siRNAs) are an important clade of regulatory small RNAs that are also capable of altering gene expression [4]. The *TAS3* locus, a well characterized conserved miR390 target, generates phased ta-siRNAs that mediated cleavage of *ARF3* and *ARF4* mRNAs [29]. After searching wheat ta-siRNAs generated from *TAS3* locus as described [39], we found that they were of very low abundance in our libraries (TPM less than 1), and their targets were not well supported by our degradome sequencing data, suggesting that these ta-siRNAs might be inactive in wheat grains. However, we also identified a long miRNA-like locus (NCBI EST No. JV867359) generating 21–22 nt phased small RNAs (Figs 2A and S3). This locus was originally identified by us as a new miRNA precursor, but then considered as a phased small RNA-related locus after investigating the distributions and numbers of unique sequences along the precursor.

As shown in Fig 2A, large numbers of reads were produced from ten 21–22 nt phases, with 5′_D sequences more abundant than 3′_D sequences on average. Unlike the *TAS3* locus where ta-siRNAs are generated from double-stranded RNA, this long miRNA-like locus produces more than 95% of the total small RNAs on one strand. Similar loci with long hairpin structures have been found in rice [16], but our identified locus has a very different sequence compared to those in rice, inferring it is first defined in wheat. Target genes could be predicted for some of the 20 phased small RNAs from both hairpin arms. Cleavage of TC438391 by 3′_D2 and Ta.88023 by 5′_D8 was verified by degradome sequencing data (Fig 2B), confirming that these small RNAs can be classified as ta-siRNAs. TC438391 is a transcript with unknown function, whereas Ta.88023 encodes a NBS-LRR resistance-like protein. Except 3′_D2 and 5′_D8, other ta-siRNAs from this locus might have unknown functions and regulate different targets in a tissue-specific manner.

### Differentially expressed miRNA families in the development of wheat grains

**Fig 3:**
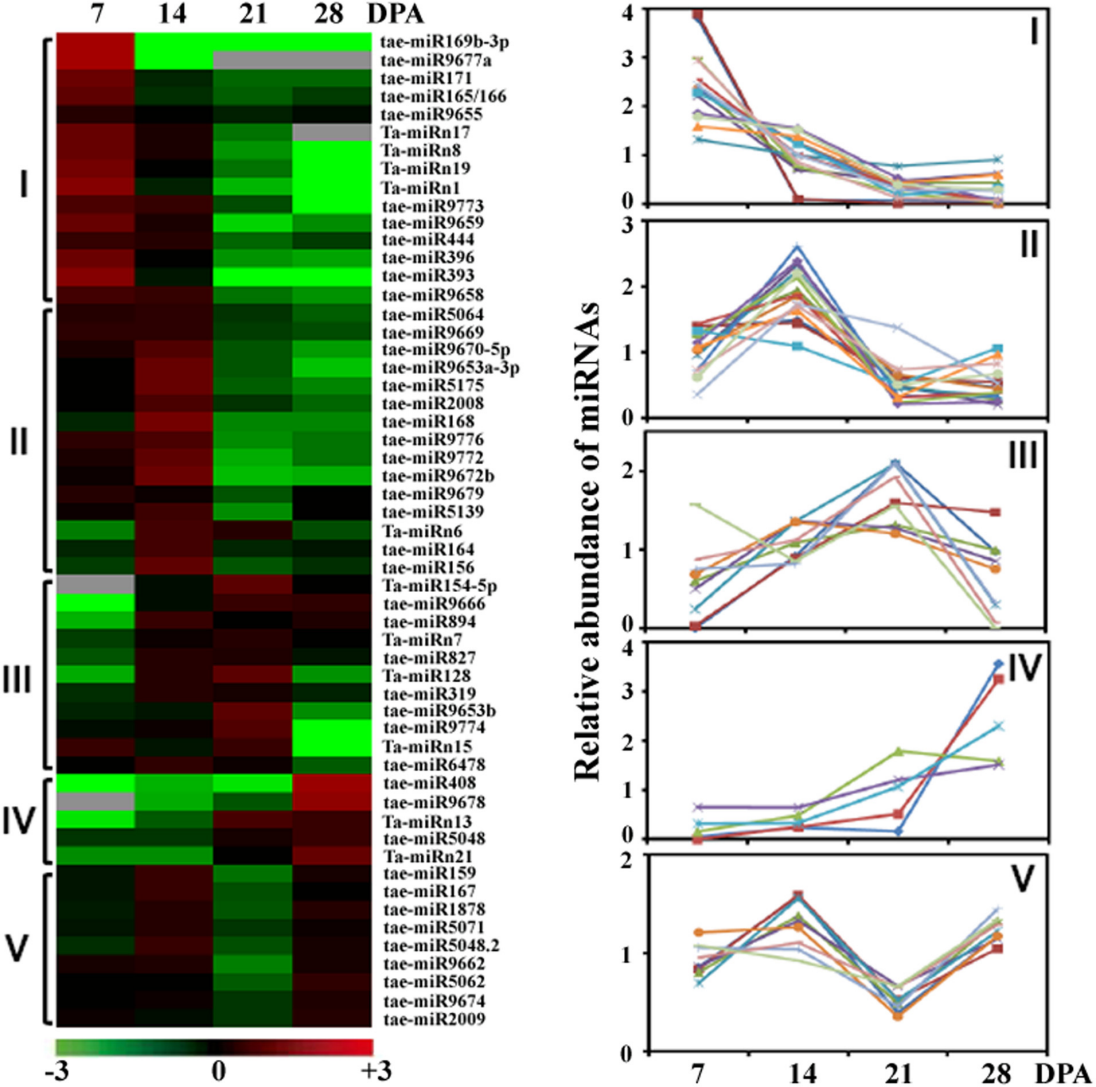
The 55 wheat grain development-related miRNAs. Hierarchical cluster analysis of 55 differentially expressed miRNAs in wheat grains (7–28 DPA) was performed by comparing miRNA abundances (TPM) in each library to the average of the four libraries. Color scale indicates fold-change as log_2_, from high (red) to low (green). Five miRNA clusters are shown on the right.

To explore the potential regulatory roles of miRNAs in grain development, we analyzed differential expression profiles of miRNA families including known and novel miRNAs. To minimize noise and improve accuracy, we selected only the miRNAs with abundances over 10 TPM in at least one library for comparison. After statistical assessment (*P* < 0.01) and cluster analysis (Fig 3), 55 miRNA families including 45 known and 10 novel miRNAs exhibited differential expression during grain development and clustered into five groups (I–V) with similar profiles. miRNAs with a single expression peak at 7 DPA, 14 DPA, 21 DPA or 28 DPA were classified as Group I, II, III or IV. Group V included miRNAs with two separate expression peaks at 14 DPA and 28 DPA. For 13 highly conserved miRNA families differentially expressed in wheat grain development (Fig 3), five (miR165/166, miR171, miR393, miR396 and miR444), three (miR156, miR164 and miR168), two (miR319 and miR827), one (miR408) and two miRNAs (miR159 and miR167) belonged to Group I, II, III, IV and V, respectively. The different expression patterns of these miRNAs indicate that they control grain development in a temporal manner. It is noted that some development-related miRNAs including miR156, miR167 and miR827 were also indentified by previous studies [18,19], demonstrating their important regulatory roles in wheat grain development.

### Targets of development-related miRNAs in wheat grains

**Fig 4:**
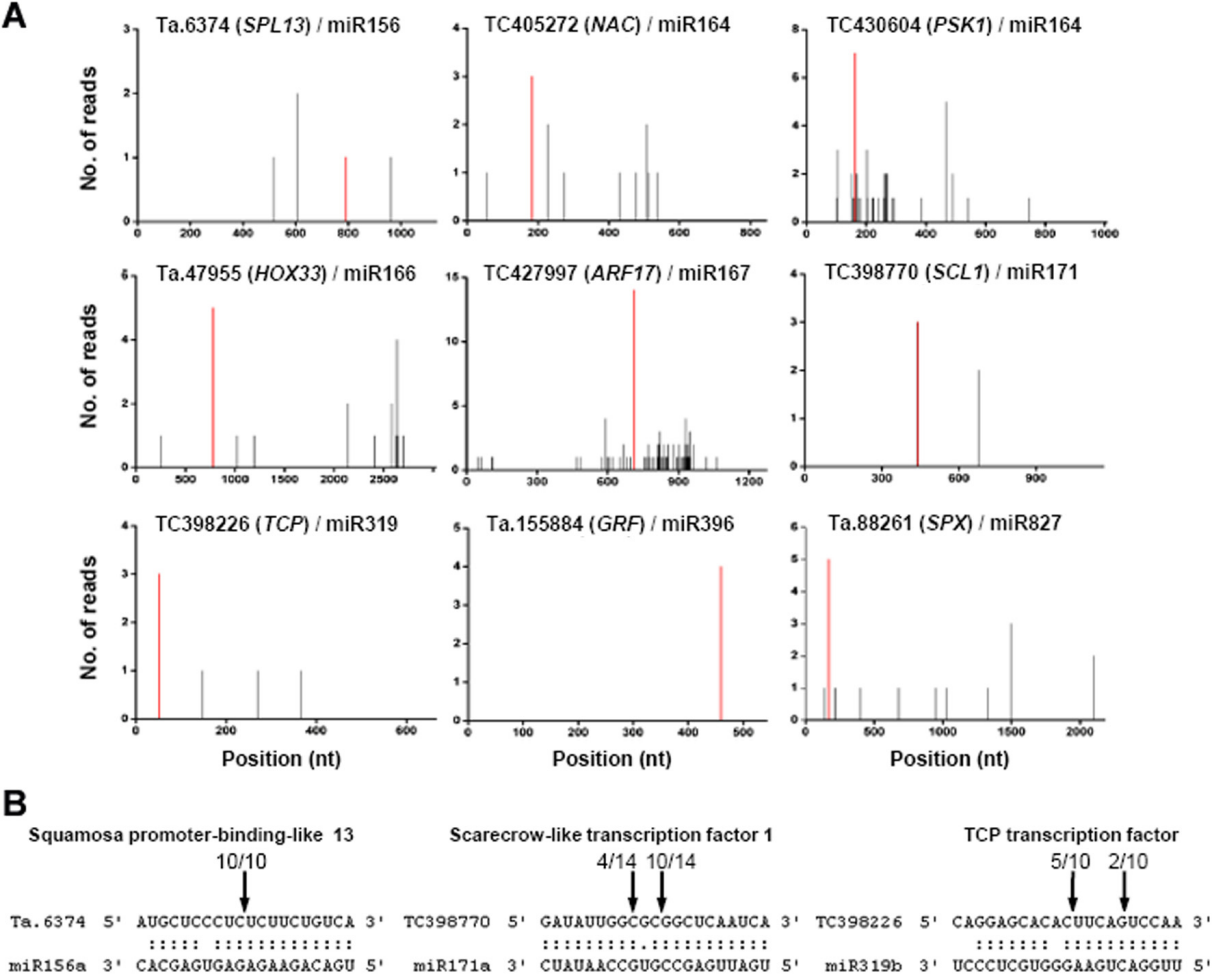
Verified targets of conserved development-related miRNAs. (A) Confirmation of target mRNA cleavage by degradome sequencing. The t-plots show sequence abundances in the position of the target transcripts, with red lines indicating sequence reads consistent with miRNA-directed cleavage. All the data of the t-plots originated from degradome library II. (B) Mapping of target mRNA cleavage sites using 5′-RACE. Arrows indicate cleavage sites and the number shows the frequency of sequenced clones.

To further investigate the functions of the 55 development-related miRNAs differentially expressed during grain development, we performed target prediction combined with degradome analysis (S6 Table). The targets for highly conserved miRNAs were mostly supported by degradome sequencing data (S6 Table). Most verified targets/miRNA modules were highly conserved among monocots and dicots, such as *SPLs*/miR156, *NACs*/miR164, *HOXs*/miR166, *ARFs*/miR167, *SCL1*/miR171, *TCPs*/miR319, *GRFs*/miR396, *SPX*/miR827 (Fig 4A). In particular, miR164 was validated to regulate a new class of targets encoding phytosulfokine-alpha 1 precursor (PSK1). This regulation is also present in rice but absent in *Arabidopsis* [40], indicating it is less conserved and emerged more recently in evolution. To further confirm the degradome data, we selected three targets of conserved miRNAs for validation using rapid amplification of 5′ cDNA ends (5′ RACE). As shown in Fig 4B, the major cleavage sites of TC398770 (*SCL1*) and TC398226 (*TCP*) were present at the canonical position, consistent with corresponding degradome data (Fig 4A). In addition, miR156-mediated cleavage of Ta.6374 (*SPL13*) mRNA, which was weakly identified by degradome data (Fig 4A), was also verified by 5′ RACE, indicating that these targets of conserved miRNAs can be well supported by degradome data combined with 5′ RACE.

**Fig 5:**
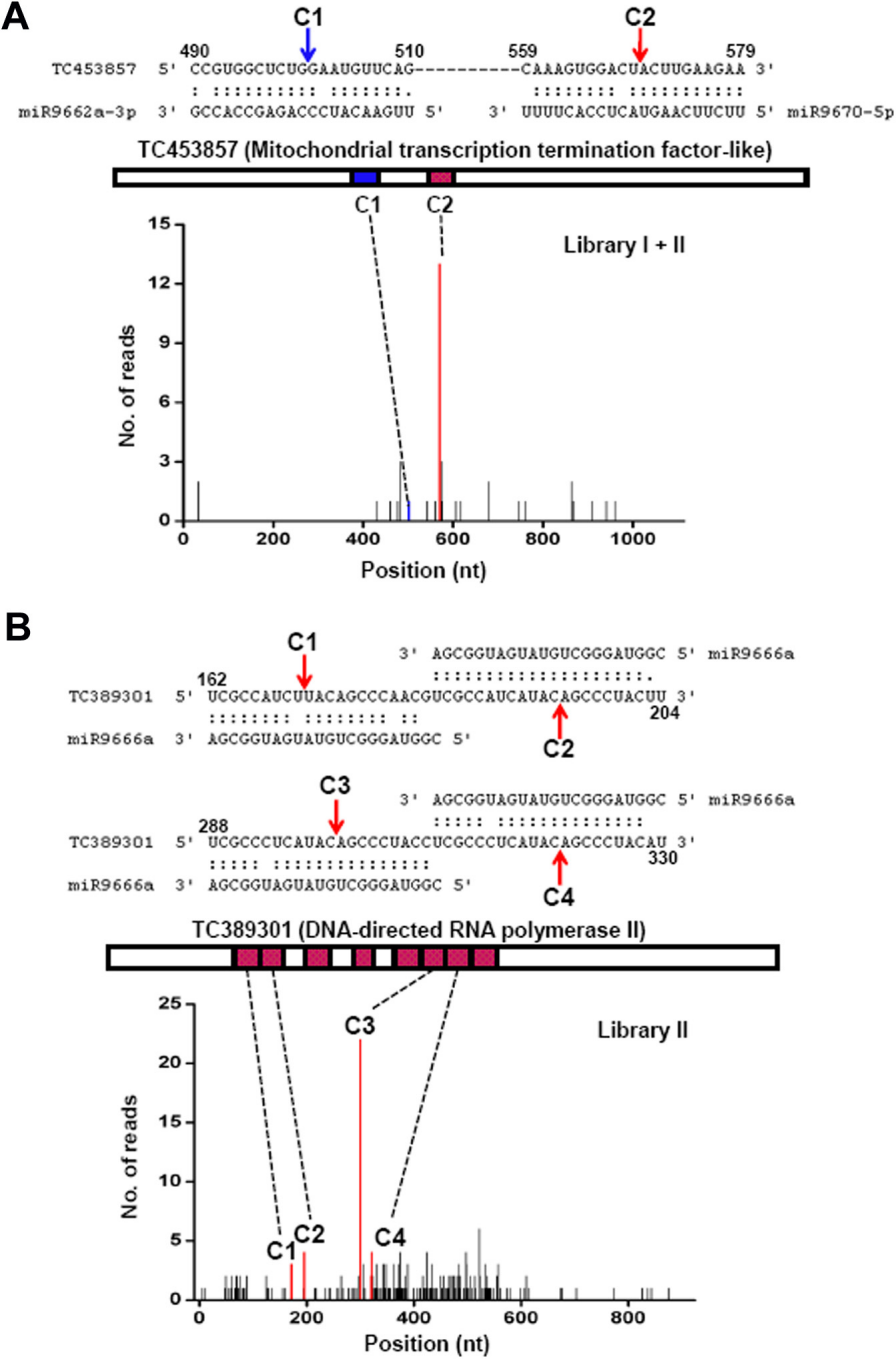
Identification of two unique targets for non-conserved development-related miRNAs. (A) A target (TC453857) was regulated by two different miRNAs. The predicted cleavage sites (C1 and C2) were shown in the blue and red boxes in the transcript, respectively. The blue or red lines in the t-plot indicate sequence abundances consistent with the C1 site or C2 site. miRNA:mRNA alignments along with C1 and C2 sites are shown above. (B) A target (TC389301) with multiple similar miRNA sites was intensively regulated by miR9666. The predicted cleavage sites are shown as red boxes in the transcript. The red lines in the t-plot indicate sequence abundances consistent with the C1–C4 sites. miRNA:mRNA alignments along with C1–C4 sites are shown above. Degradome data origins are indicated in the t-plots.

For wheat-specific miRNAs related to grain development, only a small fraction of their targets could be confirmed by degradome data (S6 Table). However, there were complex regulatory patterns present among these non-conserved miRNAs and their targets. For example, two different miRNAs, miR9662 and miR9670, were predicted to target TC453857 transcript at the C1 or C2 sites (Fig 5A). Degradome data confirmed that this target could be cleaved at each site, but the cleavage frequency was most abundant at the C2 site targeted by miR9670 (Fig 5A), indicating that some targets may be preferentially regulated by two or even more different miRNAs in a combinatorial manner. More interestingly, we also found a target with multiple nearly identical miRNA sites at different positions. As shown in Fig 5B, there are at least eight sites of miR9666 distributed in tandem or individually on the TC389301 transcript. Degradome data verified cleavage at four sites (C1–C4), and the C3 site had many more observed cleavages than other sites (Fig 5B). We supposed that multiple miRNA sites at one target might originate from insertion and/or duplication of repeat sequences in the genome. We also identified TC402663 as a major target of miR2009 in wheat grains despite as many as 4.0 mismatches, because this target had much more abundant reads in its predicted cleavage site than other targets of miR2009 (S4 Fig). It is noted that miRNAs and their variants also affect the cleavage sites of target mRNAs, as shown by similar target mRNA cleavage frequencies mediated by miR9655a and miR9655b (S4 Fig).

### Expression correlation between miRNAs and their validated targets

**Fig 6:**
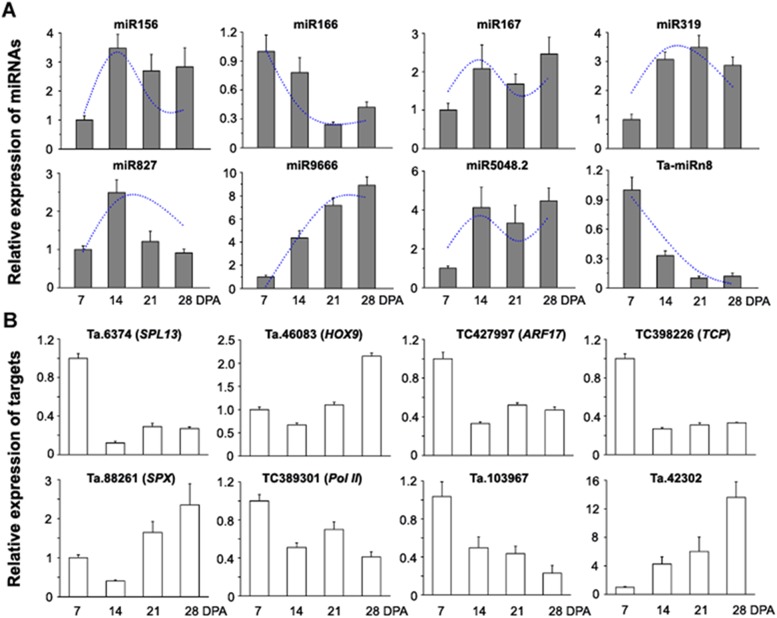
Expression correlation between development-related miRNAs and their targets. (A) qRT-PCR analysis of miRNA expression in developing grains. The dots represent the relative abundances of sequence reads in four small RNA libraries. (B) qRT-PCR analysis of target expression in the developing grains. Data represent the mean values ± SD of three replicates.

Based on sequencing data and cluster analysis (Fig 3), we selected ten development-ralated miRNAs (eight known and two new miRNAs) to detect their expression in developing grains by quantitative real-time PCR (qRT-PCR). Almost all the miRNAs determined by qRT-PCR followed similar expression trends in terms of read abundances in the libraries (Figs 6A and S5). For example, miR156 and miR164 from Group II both showed high expression levels at 14 DPA, miR166, miR393 and Ta-miRn8 from Group I showed gradual decreases in expression from 7 to 28 DPA, whereas expression of miR9666 from Group IV increased gradually from 7 to 28 DPA (Figs 6A and S5). These changes were consistent with relative changes in sequence abundance. Some minor discrepancy between qPCR and sequencing data was also observed for miR827 from Group III, which demonstrated a relatively low expression level at 21 DPA by qPCR compared with sequencing data (Fig 6A). Consistent with previous studies in wheat grains [18,19], similar expression patterns were observed for several development-ralated miRNAs such as miR156, miR167 and miR827. Overall, our results indicated that a high-throughput sequencing approach is a useful tool for profiling miRNA expression with good reproducibility.

To distinguish the effect of miRNAs on their targets in grain development, expression profiles of the validated targets of ten development-related miRNAs were also investigated by qRT-PCR. As shown in Fig 6B, the expression of *SPL16*, *ARF17*, *TCP*, *Pol II* and Ta.103967 were significantly down-regulated from 7 to 14 DPA; and conversely, during the same period, the corresponding miR156, miR167, miR319, miR9666 and miR5048.2 abundances clearly increased (Fig 6A). Similarly, expression of *HOX9*, *SPX* and Ta.42302 gradually increased from 14 to 28 DPA (Fig 6B), accompanied by down-regulation of miR166, miR827 and Ta-miRn8 expression (Fig 6A). Accordingly, these results confirmed that there was a negative correlation, in most cases, in the expression profiles of the miRNAs and their validated targets, and this was consistent with miRNA function in guiding the cleavage of target mRNAs. The one exception was miR393, whose expression was positively correlated with expression of its verified target (*TIR1*), both showing a gradual decrease from 7 to 28 DPA (S5 Fig). In addition, multiple targets of one miRNA might exhibit different expression patterns. By comparing the expression of miR164 and its validated targets, *NAC* and *PSK1* (Fig 4A), we found that the negative correlation with miR164 was more evident for *PSK1* than *NAC* (S5 Fig), inferring that *PSK1* might be a major target of miR164 in grain development. Therefore, it is possible that the negative relationship between miRNAs and some targets may be restricted in specific tissues or developmental stages, since miRNAs are not the only regulatory factors affecting their targets.

## Discussion

### A complex small RNA population present in wheat grains

Common wheat is relatively recent hexaploid (2n = 6x = 42) containing three homoeologous A, B, and D genomes of related progenitor species [41]. The wheat genome is very huge and complex due to its large amount of repetitive sequences (>80%) and its size of 17 Gb, which is more than 40 times larger than the rice genome [41]. With the advancement of wheat genome sequencing, a large number of miRNA loci (98,068) are predicted, whereas most of them are highly redundant and only very a few of them (1.7%) have evidenced expression [42], indicating that many non-conserved miRNAs undergoing fast evolution might be tissue- or cell-specific or of extremely low-abundance. In this study, our small RNA sequencing results revealed a diverse and complex small RNA population in developing wheat grains. A total of 186 known miRNAs and 37 novel miRNAs were identified in our libraries. Most new miRNAs were wheat-specific and not previously detected in other organs, suggesting that they are likely to be preferentially expressed in grains. These novel miRNAs are of high confidence due to its precise excision from precursors. More supporting evidence came from the detection of miRNA*s and sliced targets for some of them in small RNA and degradome sequencing data (Table 2 and Fig 1). Moreover, abundant miRNA variants were found in the sequencing data, and these variants originating from multiple loci may reflect the expansion of miRNA genes in wheat genome. Thus, to accurately compare miRNA abundance in different libraries, the variants with relatively high abundance can not be omitted whether they are aligned to the wheat ESTs or not.

In the small RNA libraries, known non-coding RNAs including miRNAs, rRNA, tRNA and snRNA/snoRNA account for a relatively low ratio of total small RNAs (S2 Table), whereas most small RNAs were unclassified with a length of 24 nt. The most abundant 24-nt small RNAs might be heterochromatic siRNAs which are derived from intergenic and/or repetitive genomic regions and are associated with the de novo deposition of repressive chromatin modifications [4]. Therefore, these 24-nt small RNAs are worthwhile to be explored further to distinguish their biogenesis and functions during wheat grain development. Moreover, we also identified a long miRNA-like locus generating 21~22-nt phased siRNAs (Fig 2A). 20 phased siRNAs were produced from both arms of long hairpin structure similar to miRNA precursors. Interestingly, some of these phased siRNAs have predicted targets and can cleave target mRNAs in *trans* (Fig 2B), indicating they are a new class of ta-siRNAs in wheat. Overall, our study broadens the understanding of complexity in small RNA population and their functions during wheat grain development.

### A putative functional network of development-related miRNAs in wheat grains

**Fig 7:**
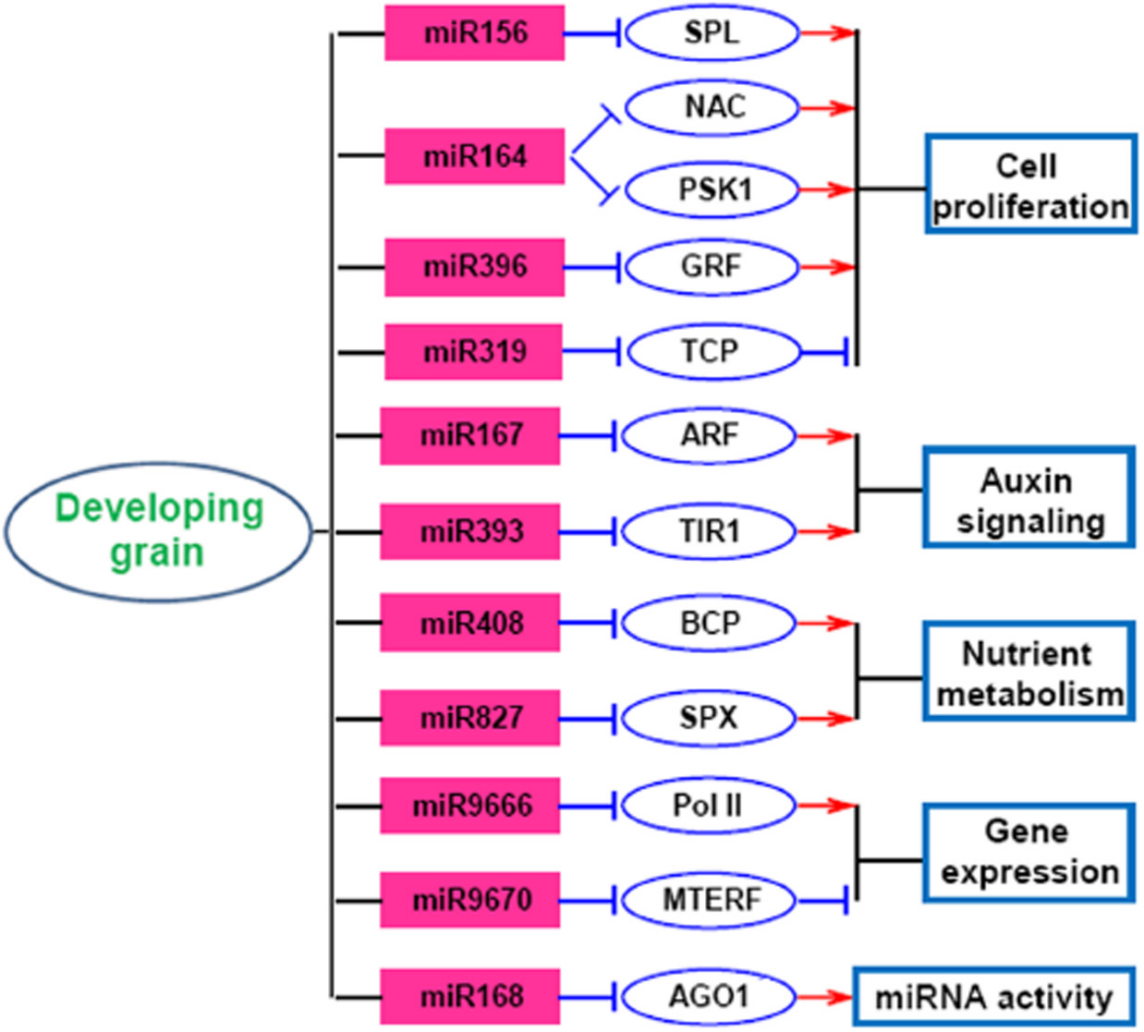
A putative miRNA regulatory network in wheat grains. The arrows indicate positive regulation and the nail shapes indicate negative regulation. PSK1, phytosulfokine-alpha 1 precursor; GRF, growth-regulating factor; ARF, auxin response factor; TIR1, transport inhibitor response 1; BCP, blue copper protein; SPX, SPX domain-containing protein; MTERF, mitochondrial transcription termination factor.

The unique biological process of wheat grain development relies on the complex regulation of gene expression. miRNAs, which are important regulators of gene expression at the transcriptional and post-transcriptional levels, have a role in the wheat grain development [18–20]. In the present study, small RNA sequencing combined with cluster analysis revealed 55 development-related miRNA families differentially expressed during wheat grain development (Fig 3). Degradome analysis confirmed that 22 miRNAs could mediate the cleavage of their targets (S6 Table). On the basis of these results, a putative miRNA-mediated regulation network was proposed in wheat grains (Fig 7).

Precise control of cell proliferation and differentiation is crucial for wheat grain development, especially at the early stages which undergo rapid increases in cell number. Consistent with this, a few development-related miRNAs are important regulators of cell proliferation. The targets of miR156 include multiple SPL transcription factors, some of which have been proved to be positive regulators of cell proliferation, resulting in increased grain size and yield in rice [10,14]. The targets of miR164 were confirmed to be NAC transcription factors and *PSK1*. In *Arabidopsis*, some NAC transcription factors can determine leaf complexity by regulating cell proliferation at the leaf margin [43]. PSK1 is a precursor of the 5-amino acid signaling peptide phytosulfokine, which can function as an autocrine-type growth factor to regulate cellular dedifferentiation and proliferation in plants [44]. The targets of miR396 were validated as GRF transcription factors. It is also reported that miR396 attenuates cell proliferation in developing leaves through repression of *GRF* activity and a decrease in the expression of cell cycle genes [11]. In addition, miR319-targeted TCP transcription factors can positively regulate the expression of miR164 and miR396, leading to reduced activities of NAC and GRF transcription factors and an inhibition of cell proliferation [11,45]. Although most targets have not displayed direct functional evidence in grain development, their conserved functions confer the likelihood that they also modulate cell proliferation in wheat grains. Consistent with this hypothesis and previous studies [18,19], the expression of these targets were relatively higher at the earlier stage of grain development, but later down-regulated sharply (Figs 6B and S5), representing a miRNA-mediated transition from rapid cell proliferation to grain filling in wheat grains.

The plant hormone auxin (primarily indole-3-acetic acid, IAA) is essential for growth and development, and controls cell polarity, cell division and cell elongation at the cellular level [46]. In *Arabidopsis*, a mutation of *AUXIN RESPONSE FACTOR 2* (*ARF2*) leads to a dramatic increase of seed size and weight [47], suggesting that auxin plays a key role in seed development. We also identified two development-related miRNAs (miR167 and miR393) involved in auxin signaling. The targets of miR167 encode ARF transcription factors, which confer auxin responses by activating or repressing auxin responsive genes in plants [48]. One target of miR167, *ARF8*, positively regulates expression of OsGH3-2, a rice IAA-conjugating enzyme, and in turn participated in the control of cellular-free IAA levels [49]. miR393 can target mRNAs encoding TIR1-like proteins, which are auxin receptors that target repressors of ARFs for ubiquitin-mediated degradation in response to auxin [50]. Interestingly, a consistent decline in expression observed for *ARF17* and *TIR1* from 7 to 14 DPA (Figs 6B and S5) may reflect a change of auxin concentration in wheat grains during this period. Hence, fine-tuning of auxin homeostasis by miRNAs and their targets is crucial for wheat grain development.

Grain filling and dry matter accumulation of wheat grains require active metabolism of nutrients including carbohydrates, storage proteins and lipids. Here, we identified two miRNAs (miR408 and miR827) associated with nutrient metabolism. The targets of miR408 encode blue copper proteins, which bind copper ions and may participate in copper transportation and storage. The target of miR827 encodes an SPX domain protein, and SPX proteins in plants are involved in internal regulation of nitrogen accumulation and phosphate homeostasis [51]. Moreover, other development-related miRNAs are regulators of gene transcription and miRNA activities. Both miR9666 and miR9670 are wheat-specific miRNAs and target mRNAs encoding a DNA-directed RNA polymerase II (Pol II) and a mitochondria transcription termination factor (MTERF), respectively (Fig 5). As is well known, Pol II catalyzes the transcription of DNA to synthesize precursors of mRNA and most snRNA and microRNA [52]. MTERF participates in attenuating transcription from the mitochondrial genome or has other functions such as intron splicing in plants [53]. For regulation of miRNA activity, miR168-targeted AGO1 is responsible for loading miRNAs and acts as an RNA slicer, determining the cleavage efficiency of miRNA targets [54]. Together, these miRNAs and their targets comprise of a complex functional network during grain development.

## Supporting Information

S1 Fig Size distribution of redundant and unique reads in four small RNA libraries. (PDF)

S2 Fig Precursors of 15 known and 37 new miRNAs. (PDF)

S3 Fig Sequence and structure of the miRNA-like long hairpin locus. (PDF)

S4 Fig Validated targets of miR2009 and miR9655 by degradome sequencing. (PDF)

S5 Fig Expression patterns of mi164, miR393 and their targets during grain development. (PDF)

S1 Table Primers used in this study. (DOCX)

S2 Table Distribution of sequence reads in the four small RNA libraries. (DOCX)

S3 Table Known miRNAs in the miRBase identified in the four libraries. (DOCX)

S4 Table Other reported wheat miRNAs identified in the four libraries. (DOCX)

S5 Table Predicted and verified targets of novel miRNAs. (DOCX)

S6 Table Predicted and verified targets of differentially expressed miRNAs. (DOCX)
