# Supplementary figures and images for: Small RNA and Degradome Sequencing Reveal Complex Roles of miRNAs and Their Targets in Developing Wheat Grains
Source: PLoS One. 2015 Oct 1;10(10):e0139658. doi: 10.1371/journal.pone.0139658 (PMC4591353; doi:10.1371/journal.pone.0139658)

**S1 Fig. Size distribution of redundant (A) and unique (B) reads in four small RNA libraries.**

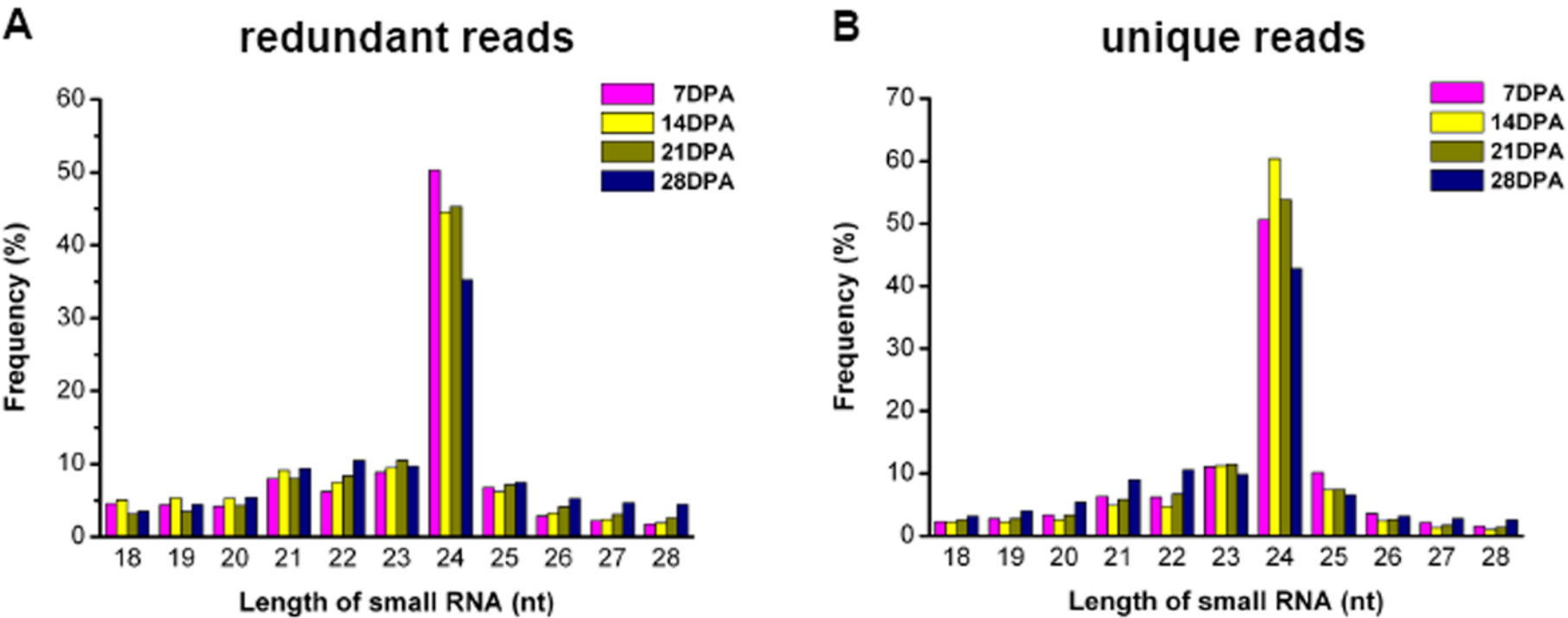

Supplement: S1 Fig — (PDF) [file pone.0139658.s001.pdf]

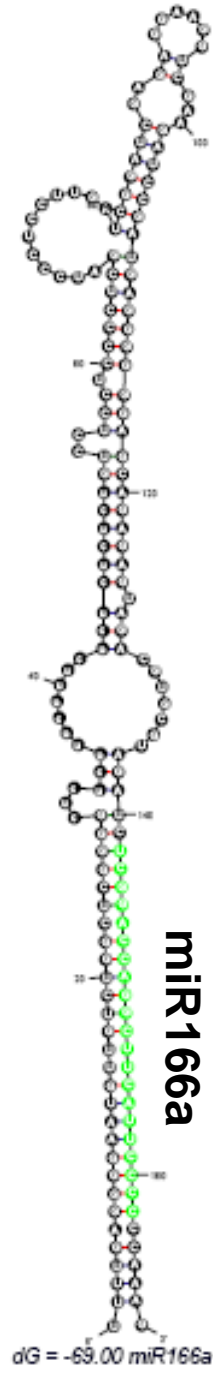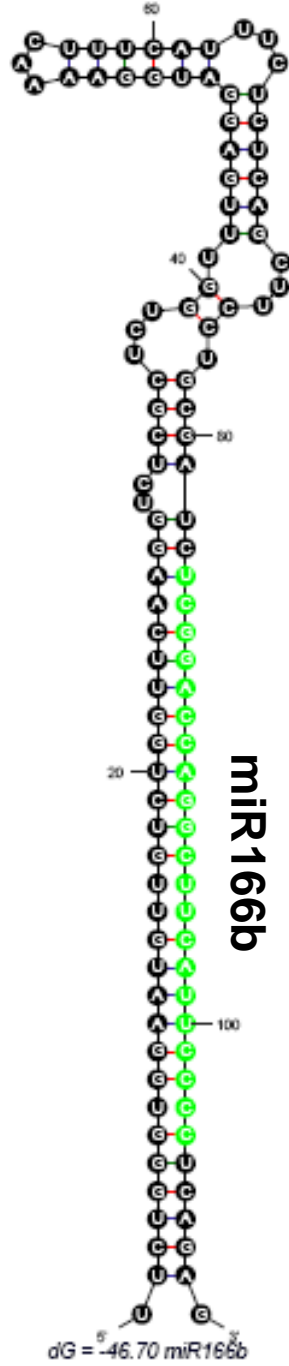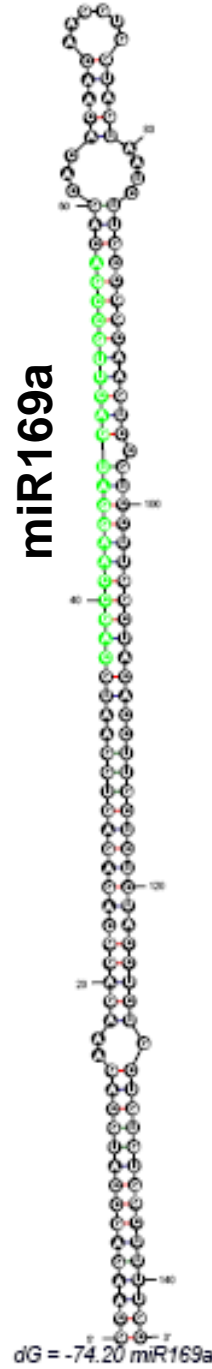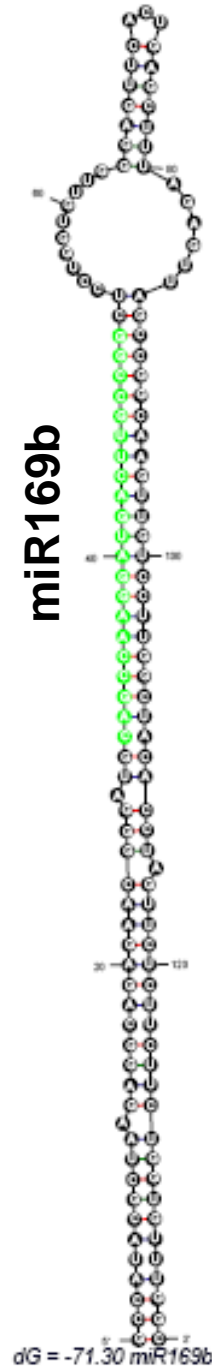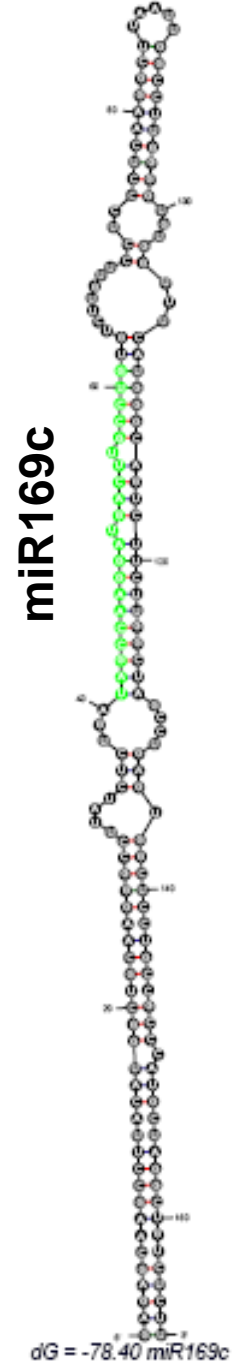

**miR393**

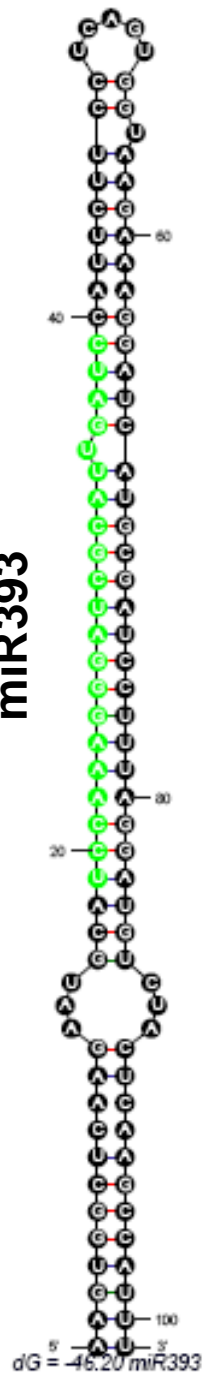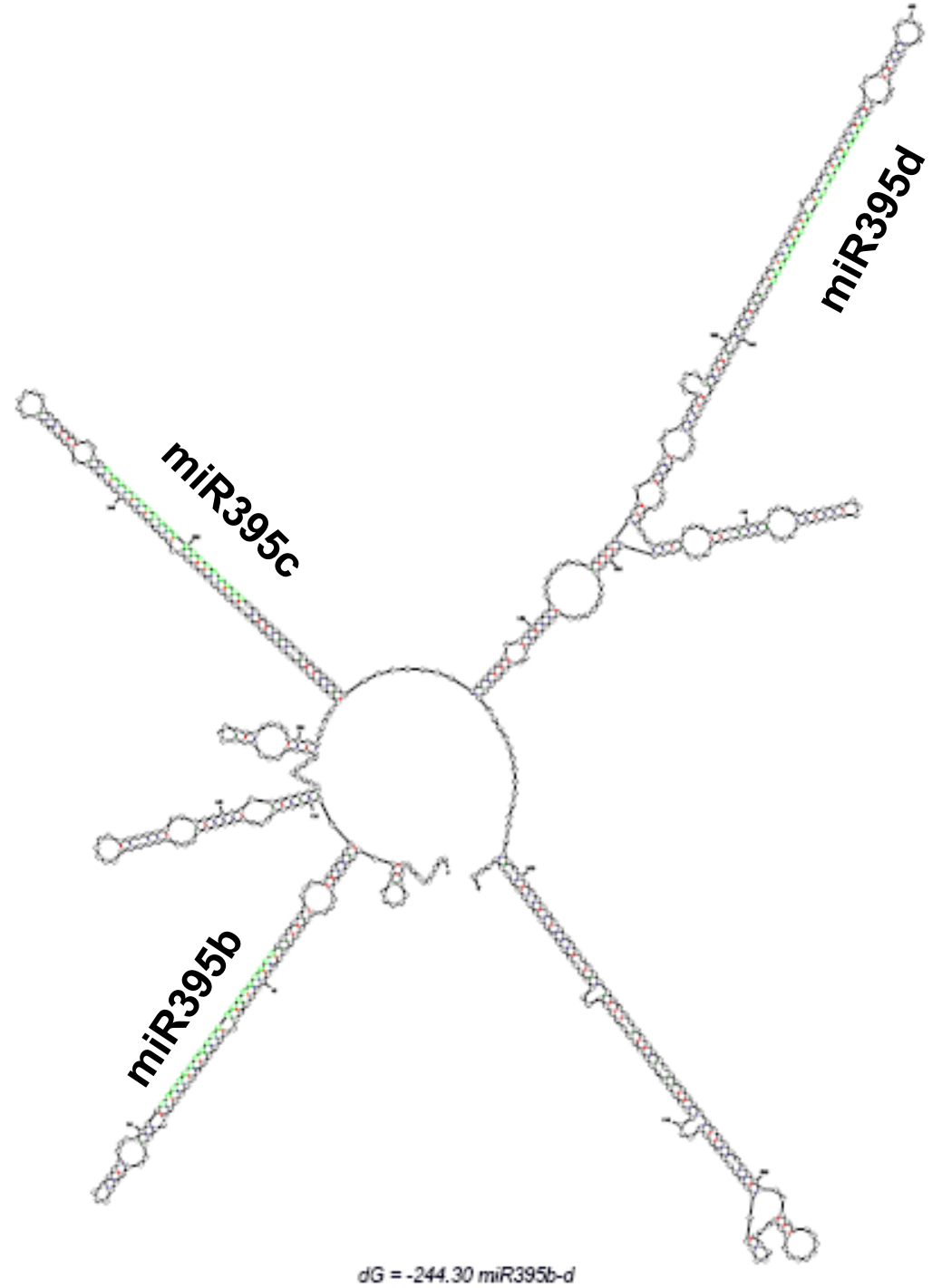

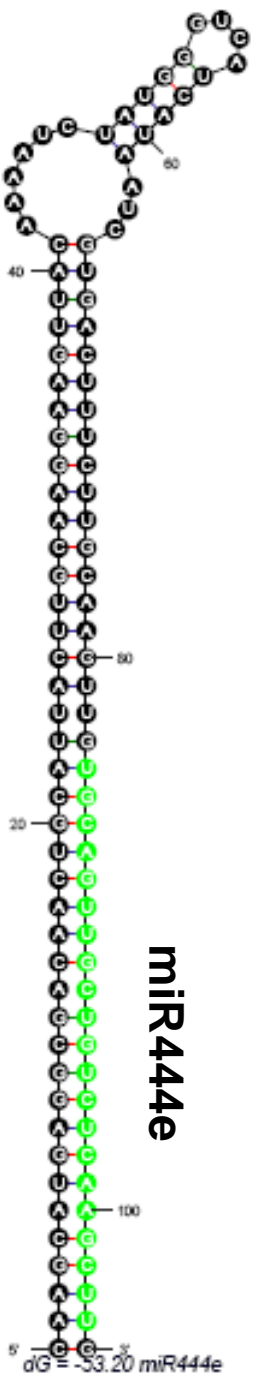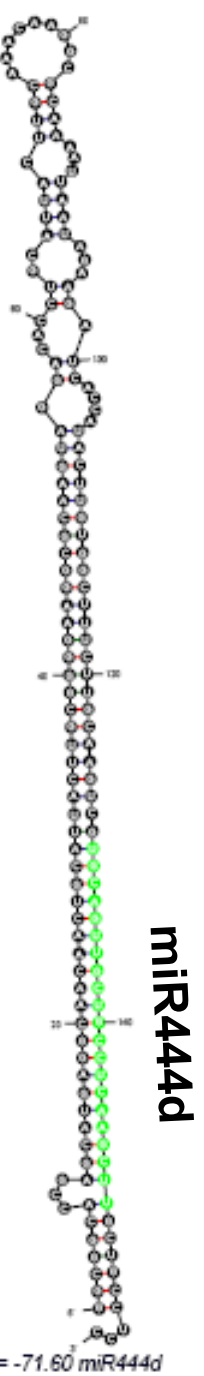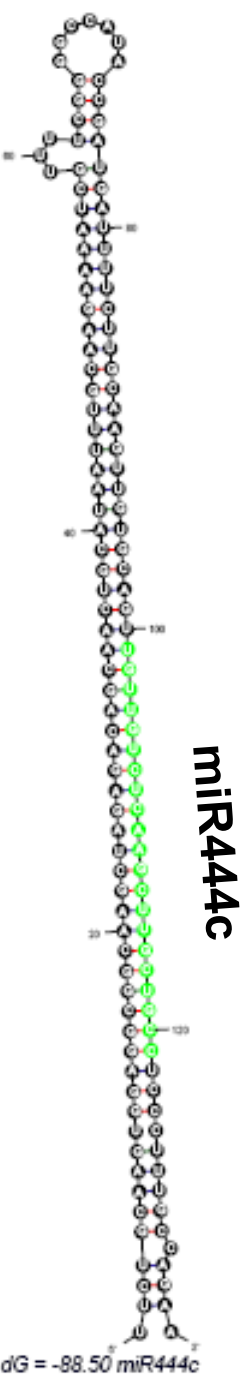

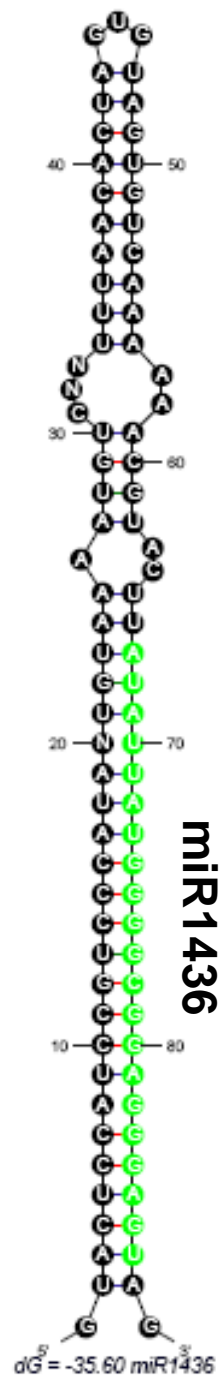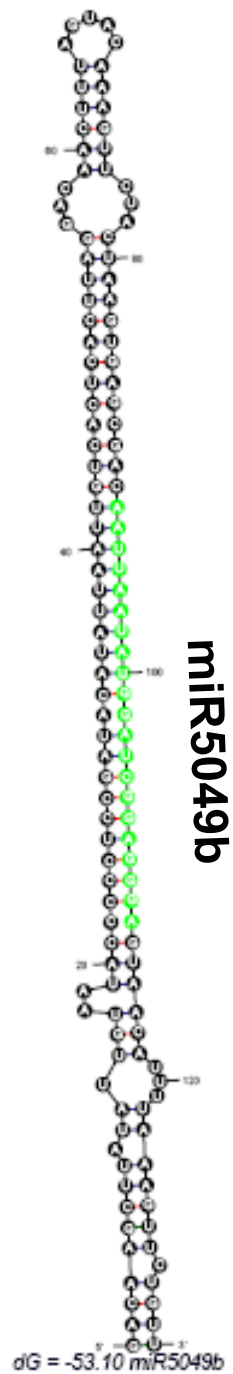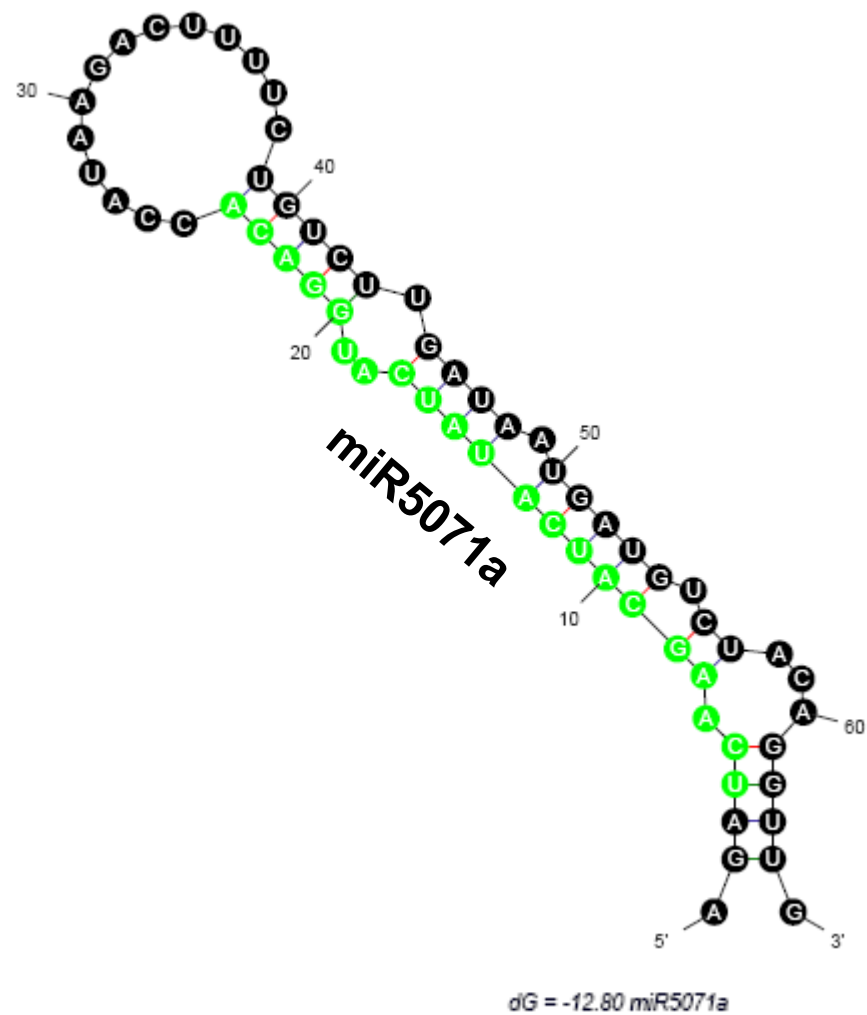

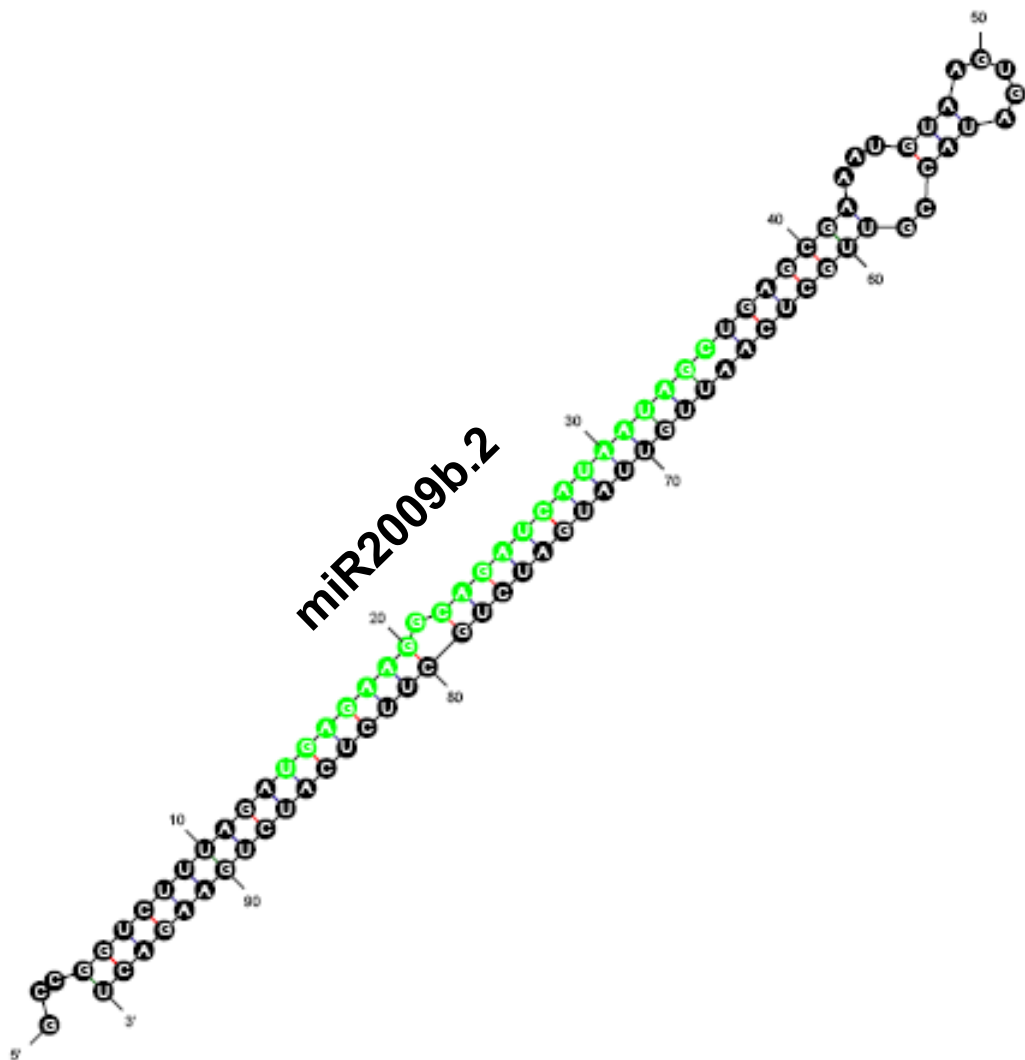

dG = -50.50 miR2009b 2

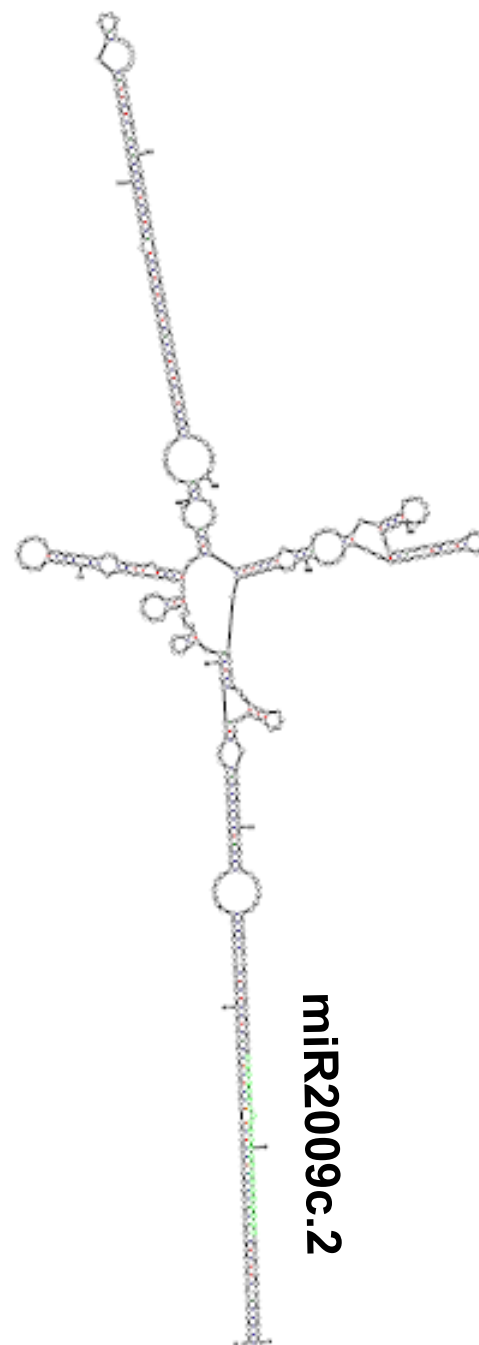
$$dG = -147.60 \text{ miR2009c } 2$$

## miR5048.2

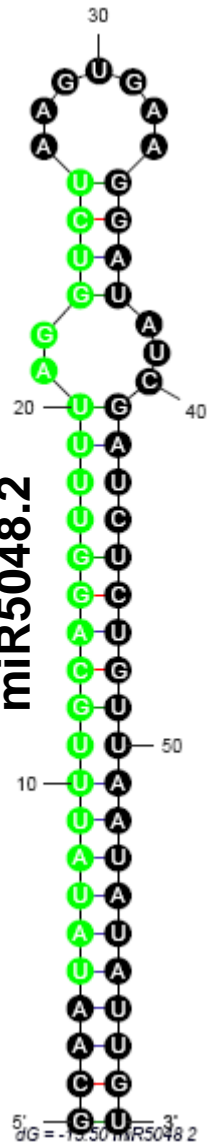

## miR5175b

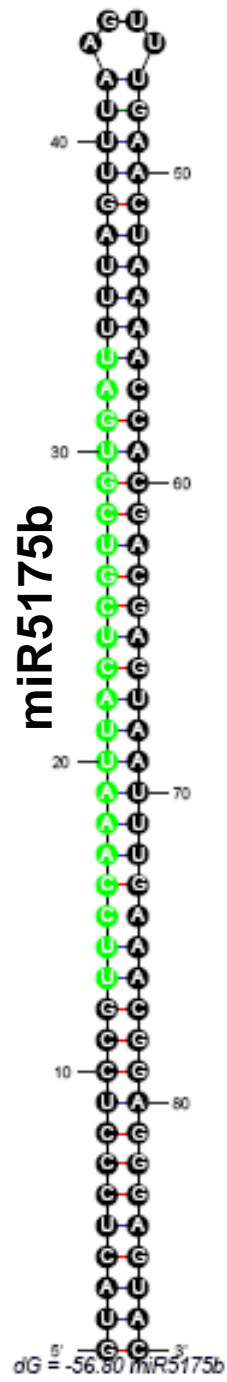

## miR5175c

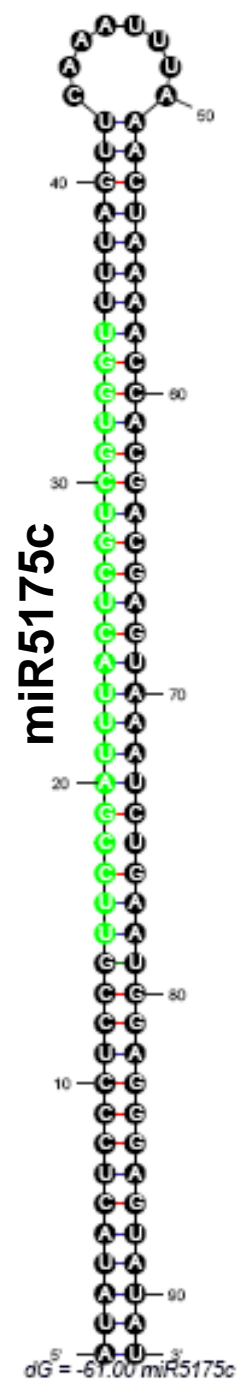

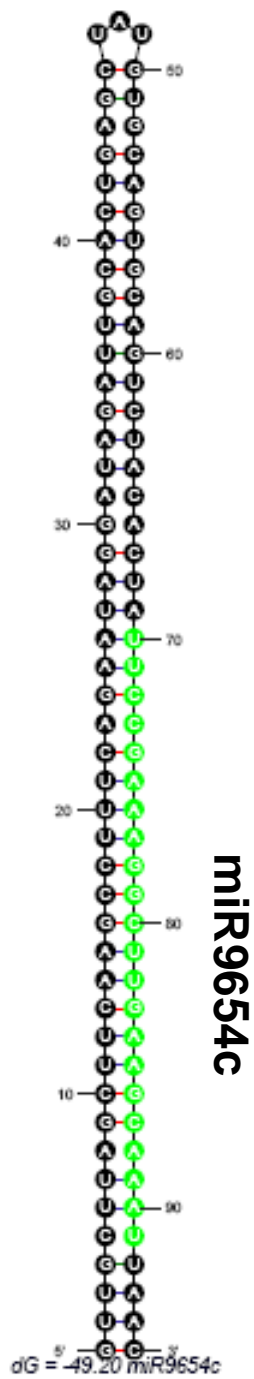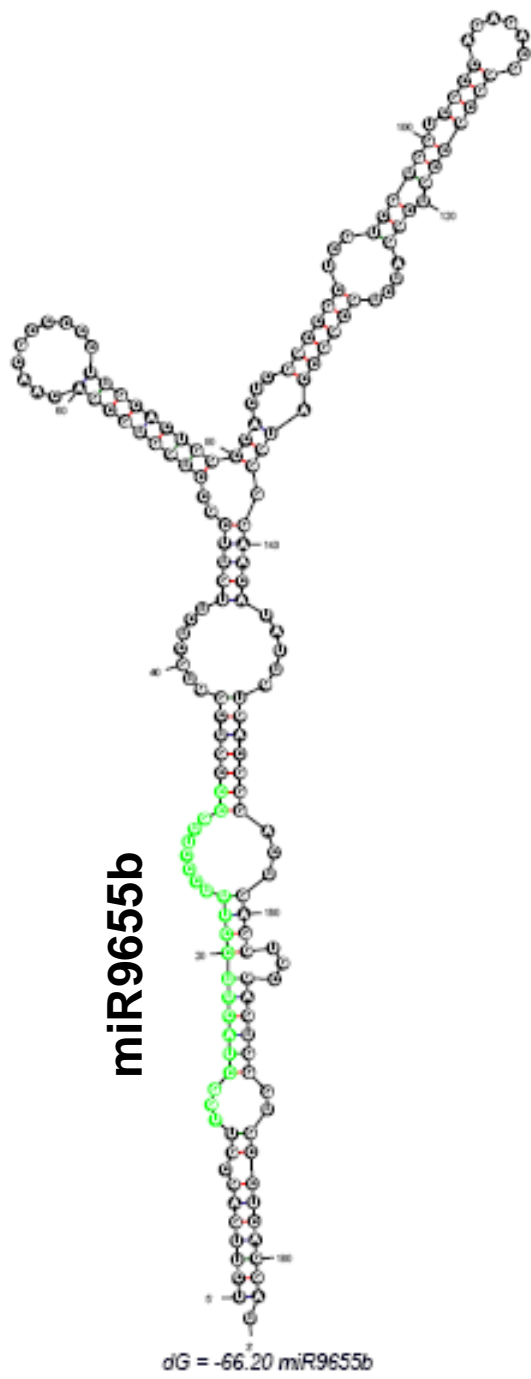



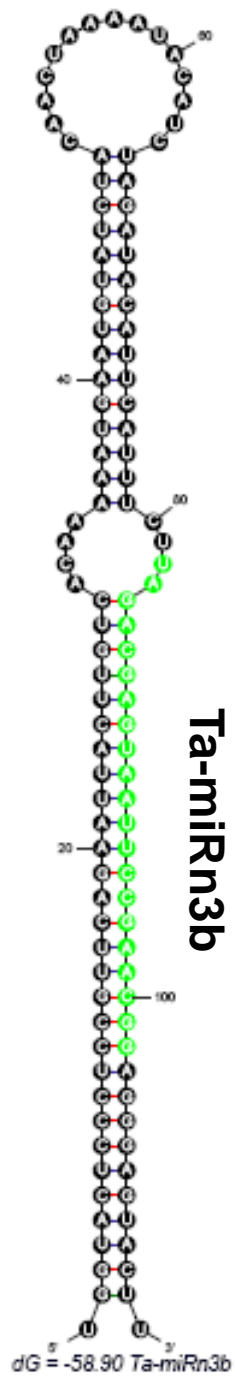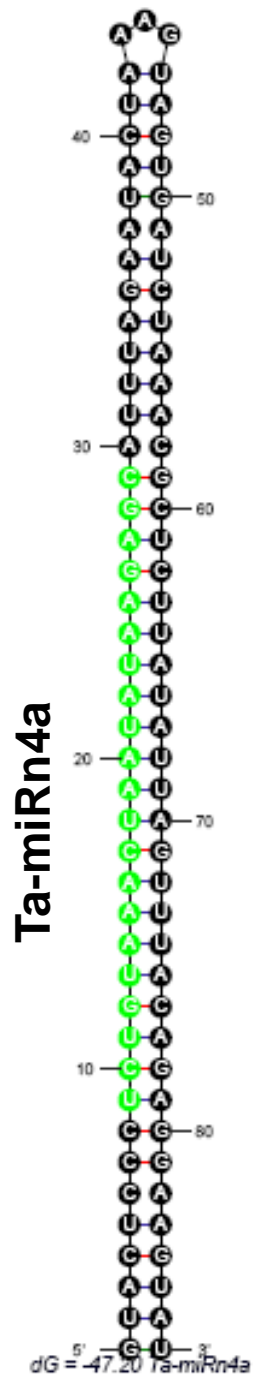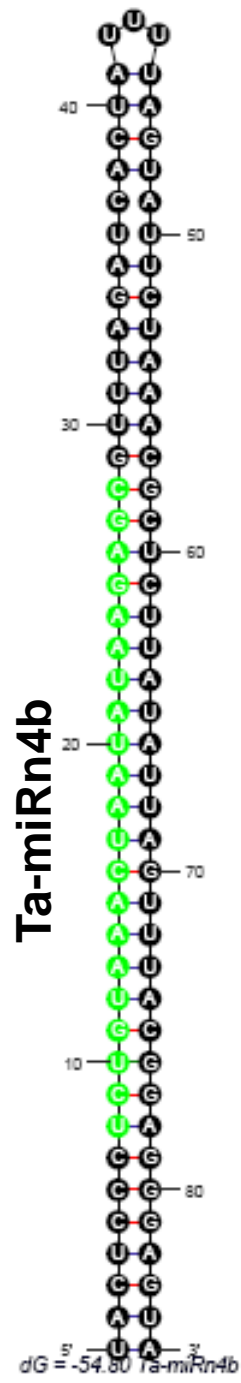

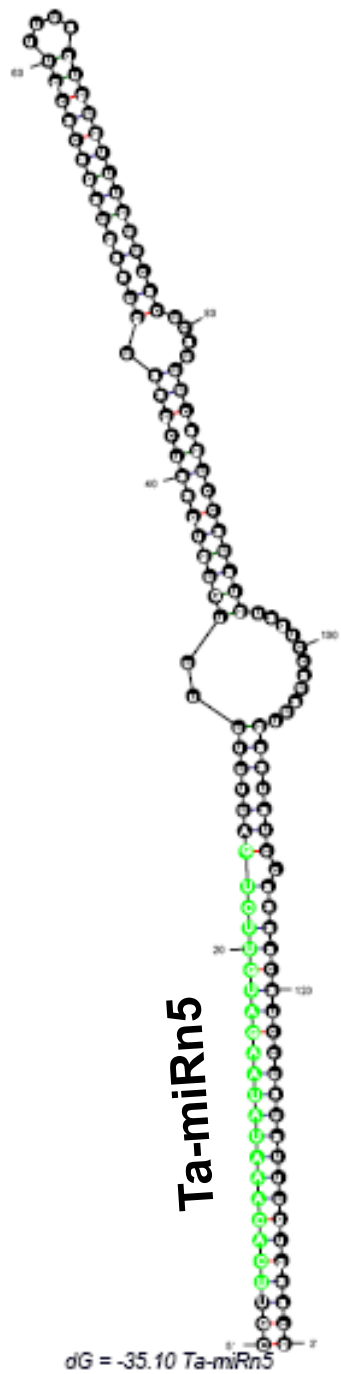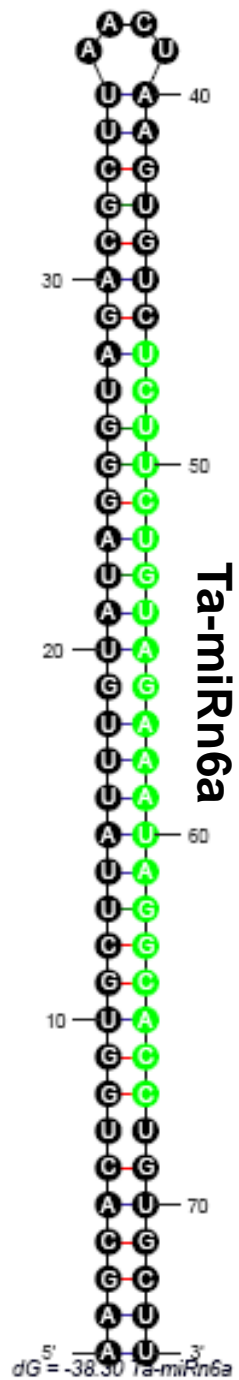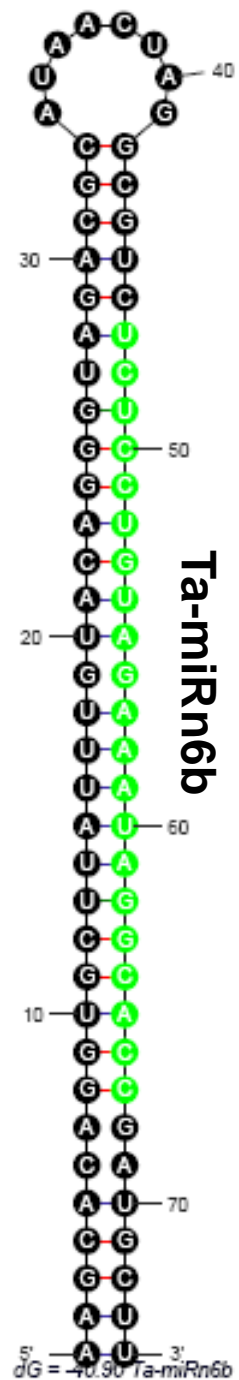

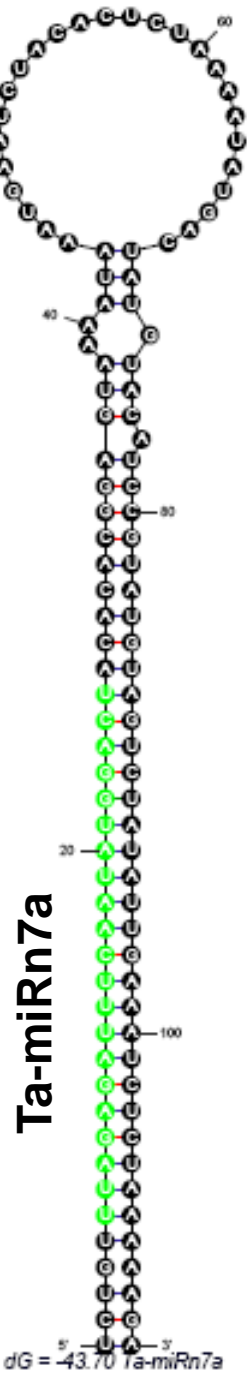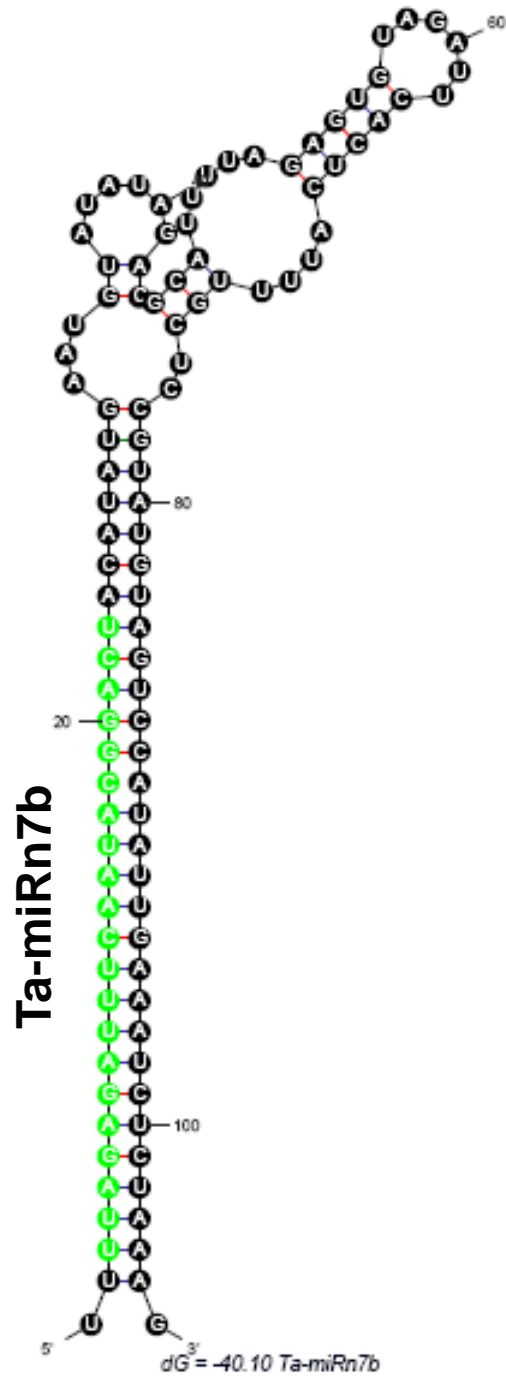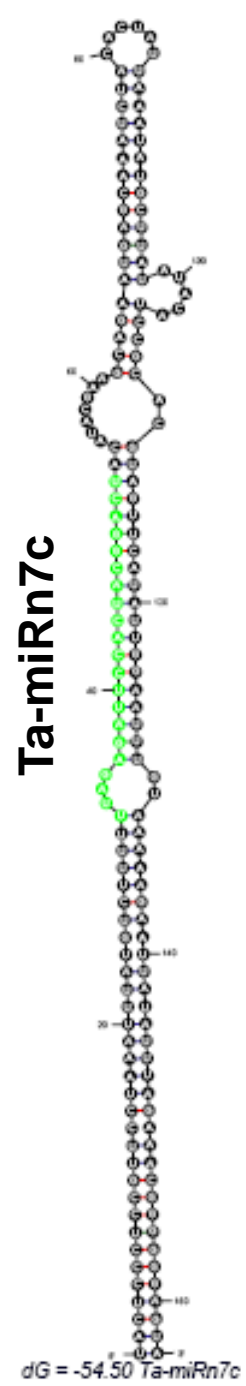

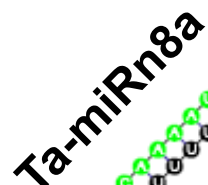

## Ta-miRn8b

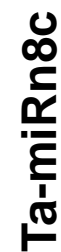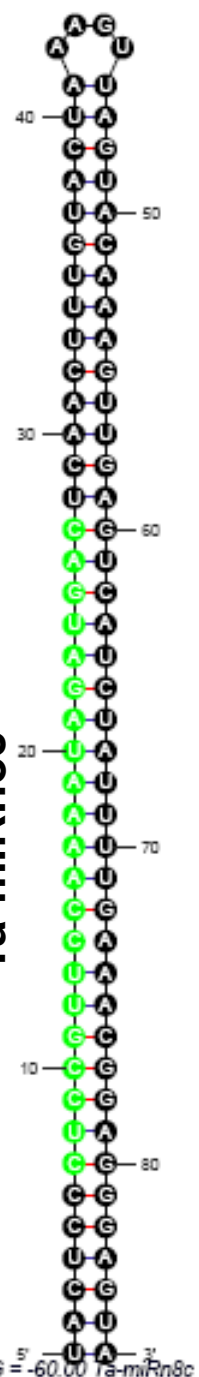

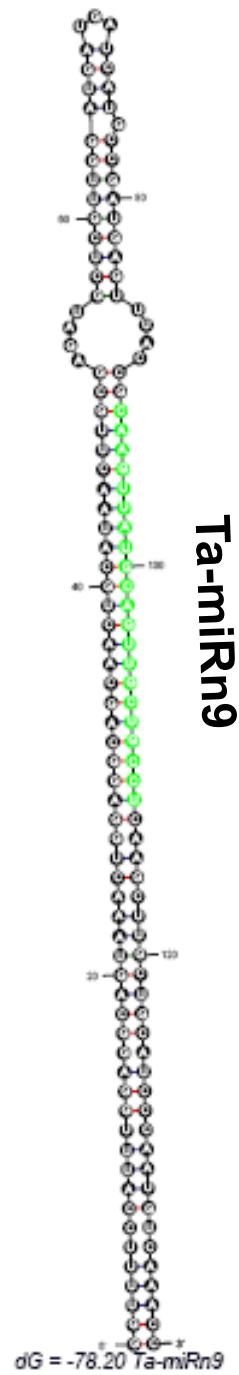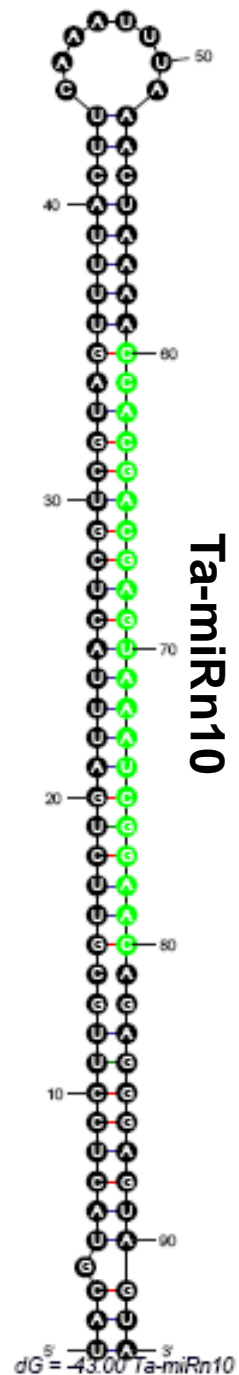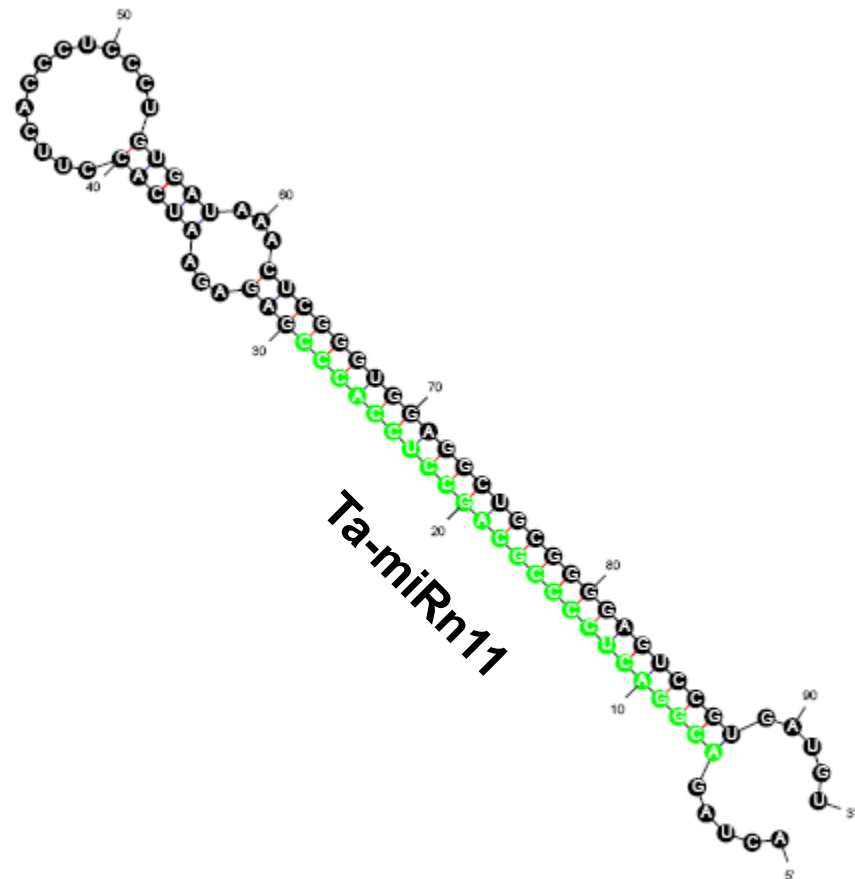

## Ta-miRn12

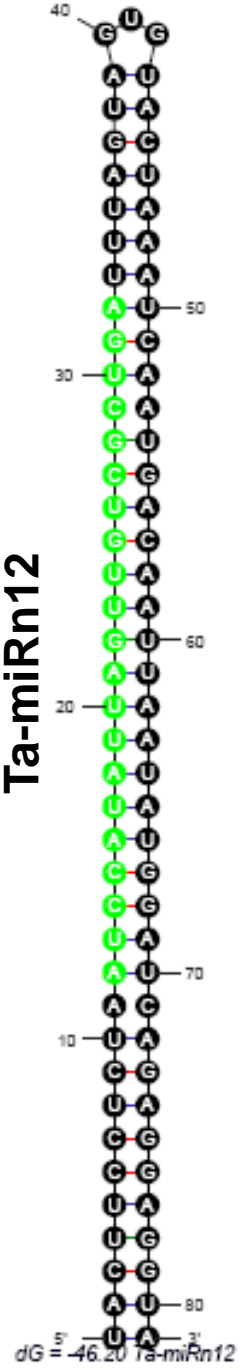

## Ta-miRn13a

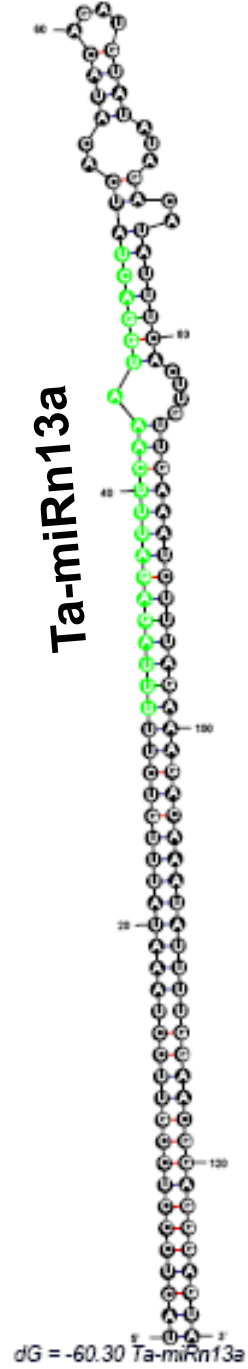

## Ta-miRn13b

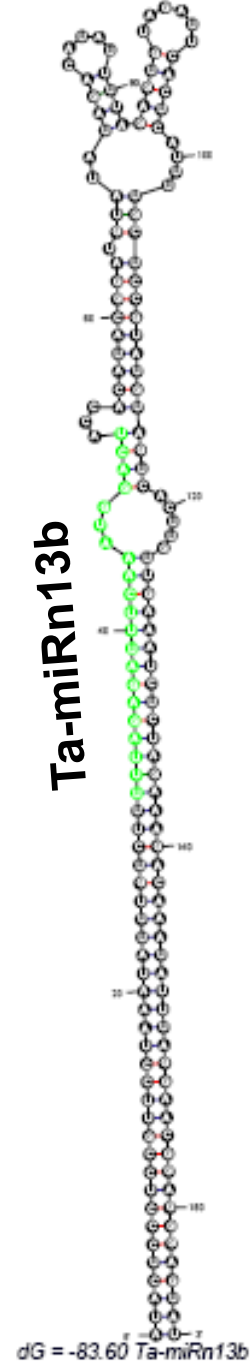

## Ta-miRn14

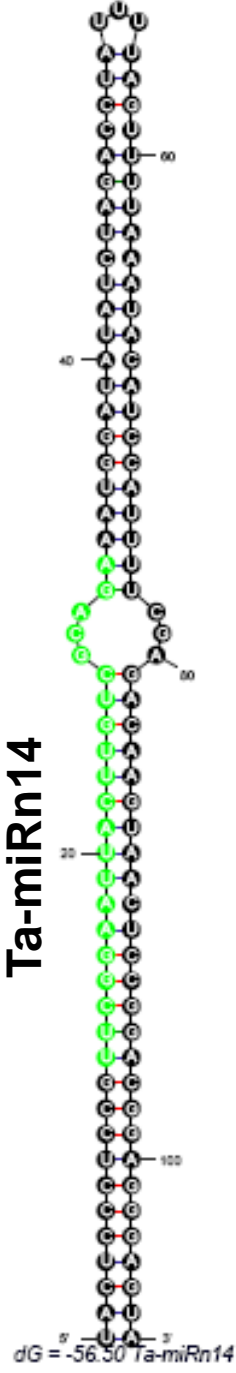

## Ta-miRn15

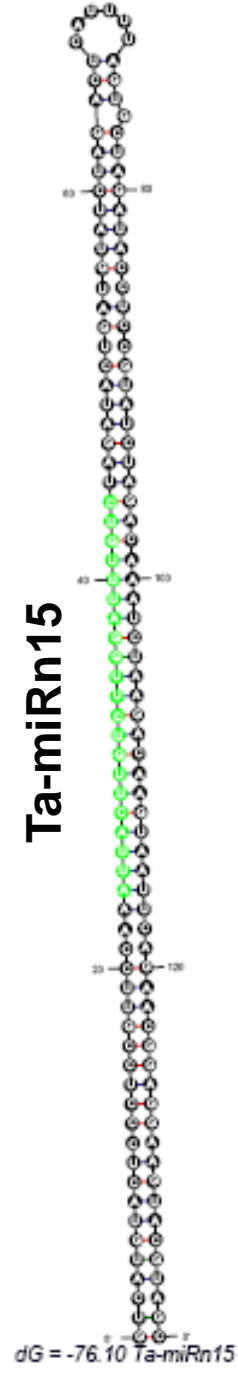

## Ta-miRn16

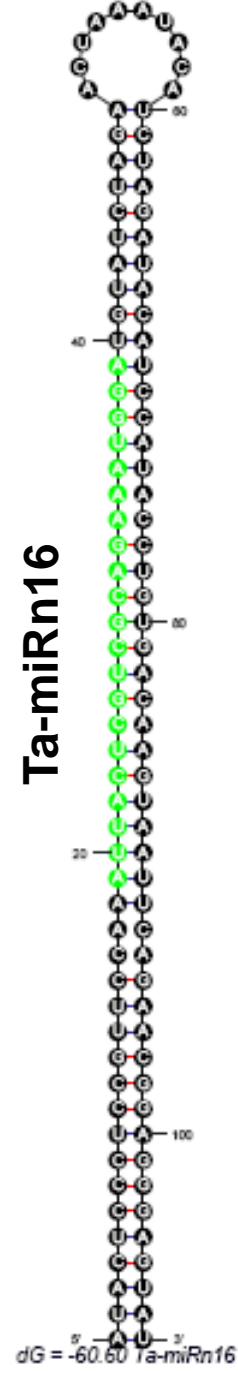

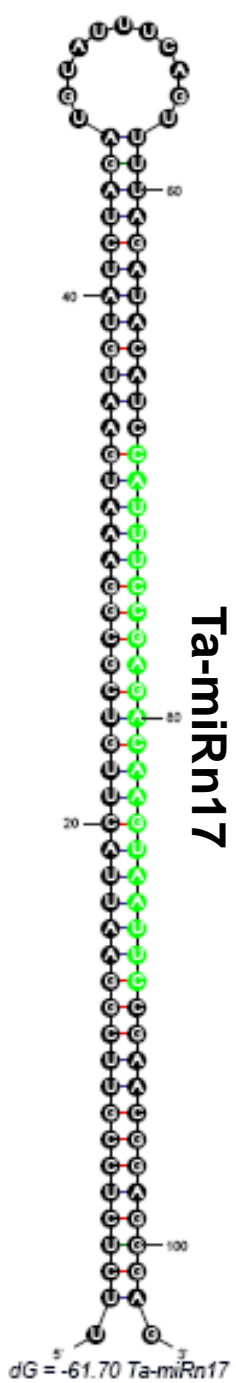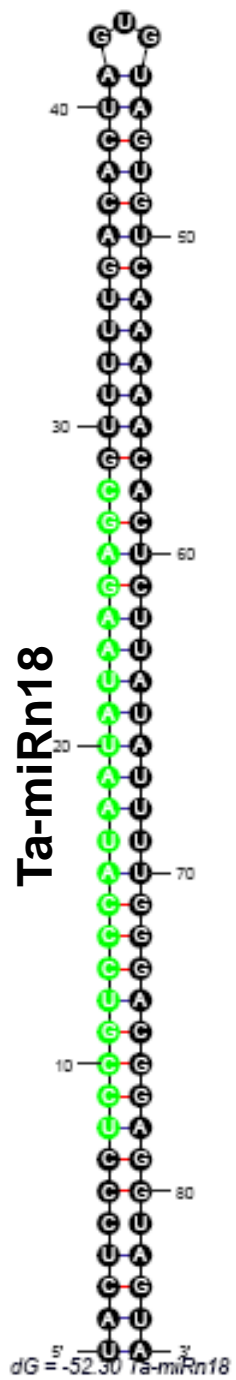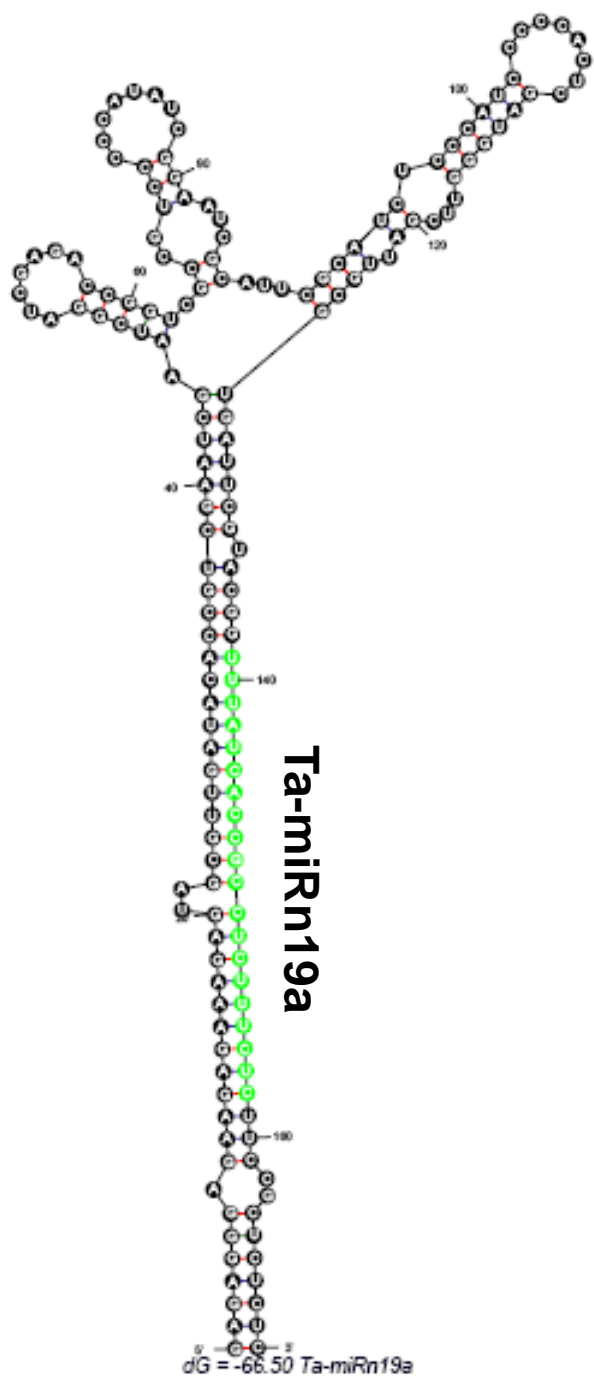

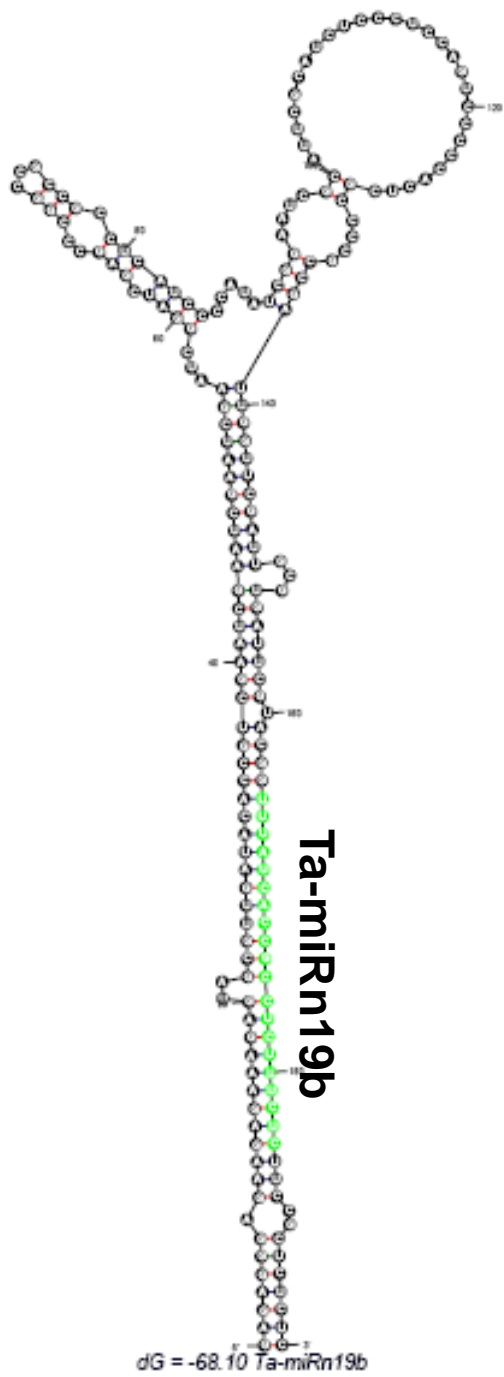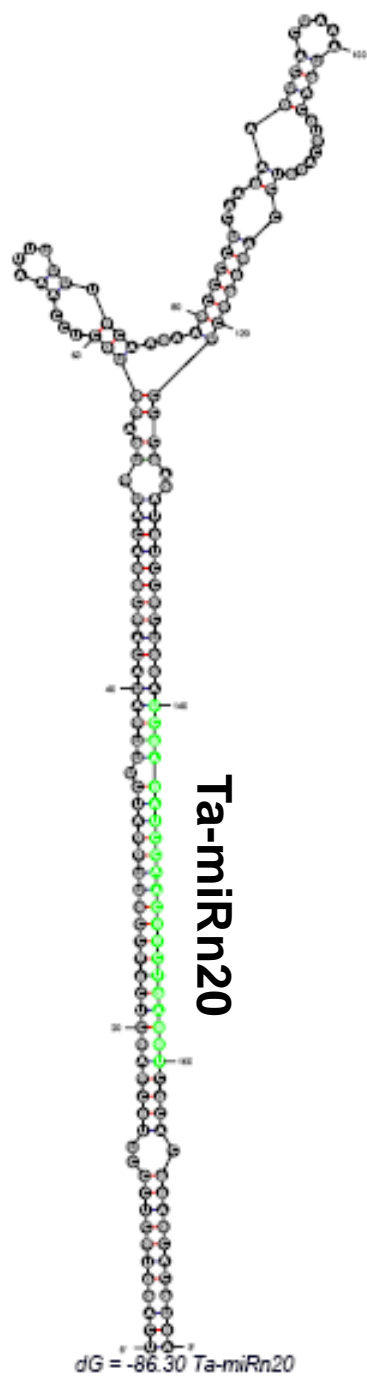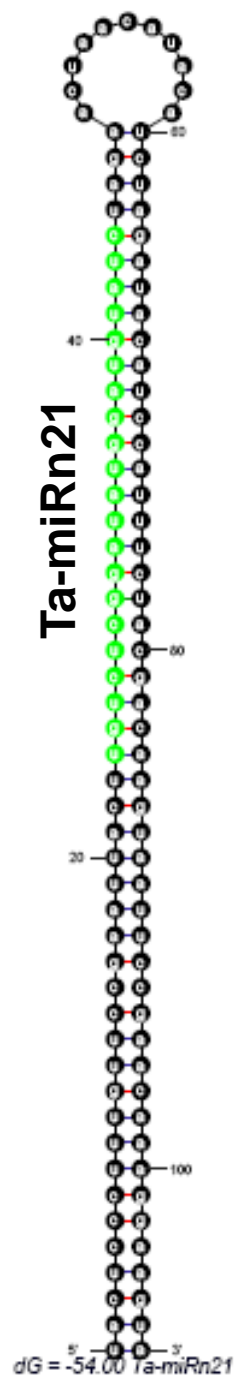

Supplement: S2 Fig — (PDF) [file pone.0139658.s002.pdf]

**S3 Fig. Sequence and structure of the miRNA-like long hairpin locus (EST No. JV867359).**

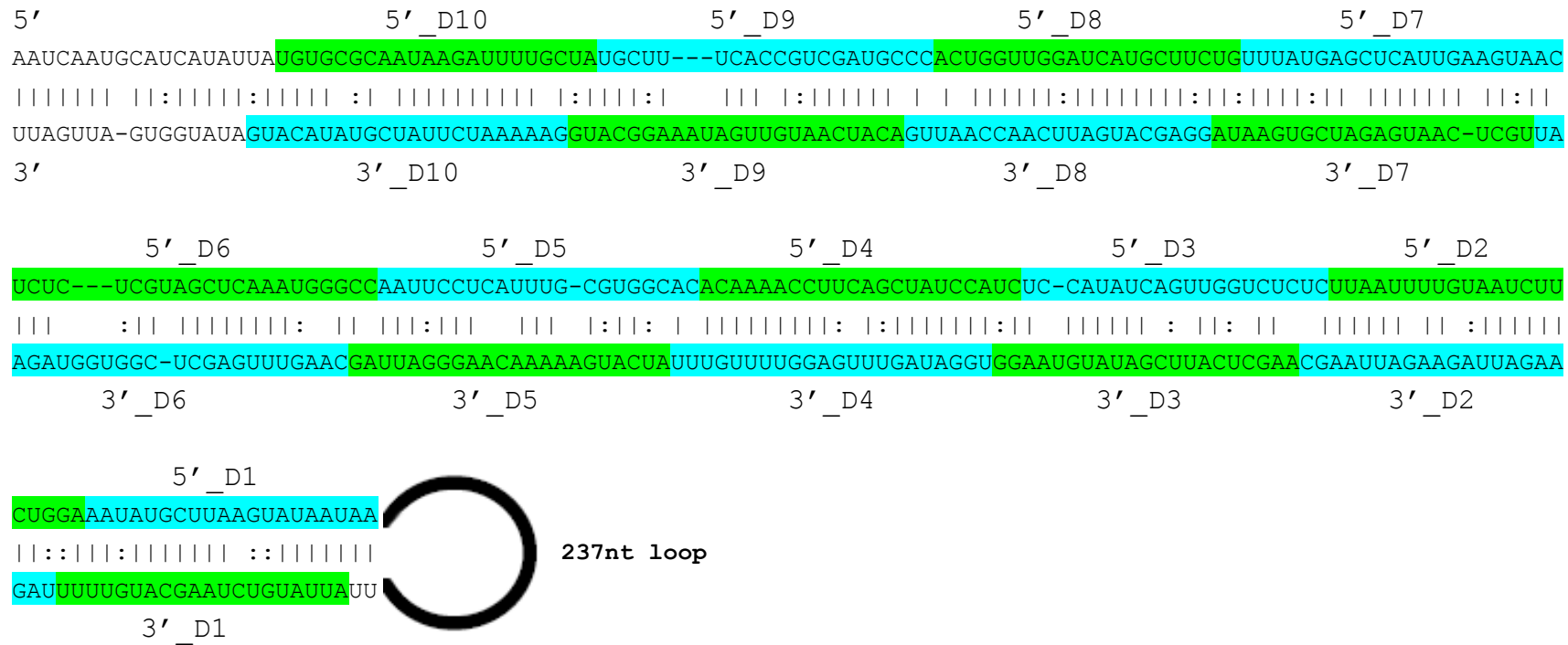

Supplement: S3 Fig — (PDF) [file pone.0139658.s003.pdf]

**S5 Fig. Expression patterns of mi164, miR393 (A) and their targets (B) during grain development.**

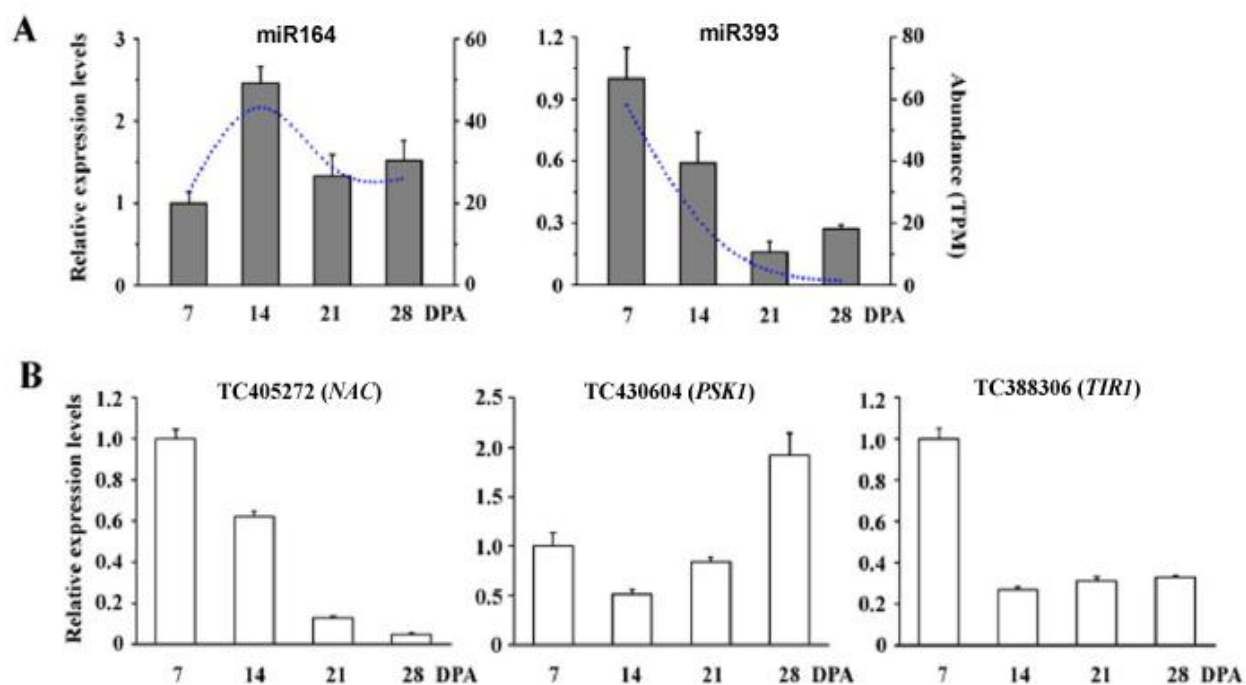

Supplement: S5 Fig — (PDF) [file pone.0139658.s005.pdf]
