# Supplementary material for: Small RNA and Degradome Sequencing Reveal Complex Roles of miRNAs and Their Targets in Developing Wheat Grains
Source: PLoS One. 2015 Oct 1;10(10):e0139658. doi: 10.1371/journal.pone.0139658 (PMC4591353; doi:10.1371/journal.pone.0139658)

S4 Fig. Validated targets of miR2009 (A) and miR9655 (B) by degradome sequencing.

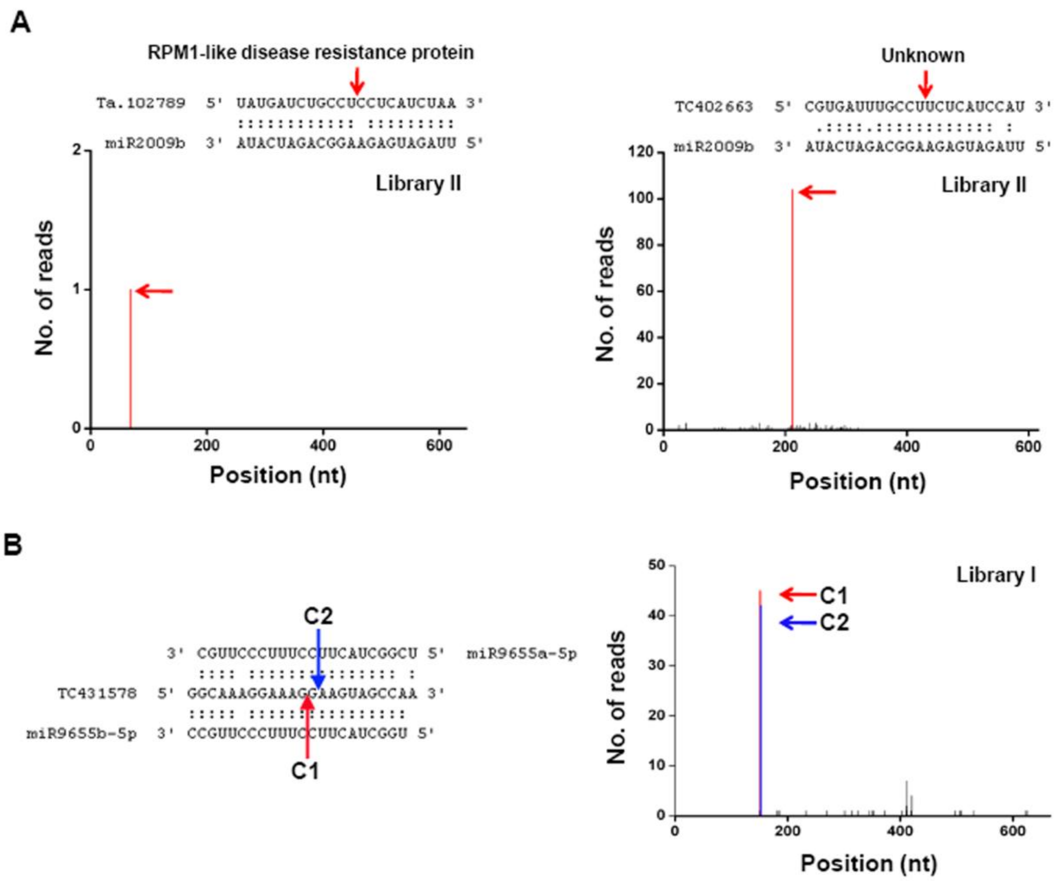

Supplement: S4 Fig — (PDF) [file pone.0139658.s004.pdf]
