# Supplementary material for: Small RNA and Degradome Sequencing Reveal Complex Roles of miRNAs and Their Targets in Developing Wheat Grains
Source: PLoS One. 2015 Oct 1;10(10):e0139658. doi: 10.1371/journal.pone.0139658 (PMC4591353; doi:10.1371/journal.pone.0139658)
Supplement: S1 Table — (DOCX) [file pone.0139658.s006.docx]

**S1 Table. Primers used in this study.**

| **Primers for 5′ RACE mapping of mRNA cleavage sites (5'-3')** | |
| --- | --- |
| Ta.6374 (*SPL13*) outer primer | CCCGTTCTGAGCAATCCTTA |
| Ta.6374 (*SPL13*) inner primer | GCCACAAATTATCCCAGCACA |
| TC398770 (*SCL1*) outer primer | CACCAACCGAAGGATTACAGC |
| TC398770 (*SCL1*) inner primer | TCGTCGCCAGTAGGAGAAATG |
| TC398226 (*TCP*) outer primer | AACATTTGGTTTCCGATCACAA |
| TC398226 (*TCP*) inner primer | TTATGTCTTCCTTTCCTCCTTGA |
| **Primers for miRNA qRT-PCR^#^ (5'-3')** | |
| miR156(b) forward primer | TTGACAGAAGAGAGTGAGCAC |
| miR164 forward primer | TGGAGAAGCAGGGCACGTGCA |
| miR166(a) forward primer | TCGGACCAGGCTTCATTCCCC |
| miR167(d) forward primer | TGAAGCTGCCAGCATGATCTGA |
| miR319(b) forward primer | TTGGACTGAAGGGTGCTCCCT |
| miR393 forward primer | TCCAAAGGGATCGCATTGATC |
| miR827 forward primer | TTAGATGACCATCAGCAAACA |
| miR9666(a) forward primer | CGGTAGGGCTGTATGATGGCGA |
| miR5048.2 forward primer | TATATTTGCAGGTTTTAGGTCT |
| Ta-miRn8 forward primer | CTCCGTTCCAAAATAGATGAC |
| **Primers for target qRT-PCR (5'-3')** | |
| Ta.6374 (*SPL13*) forward | GCTGAGCCATTTCCAGATCA |
| Ta.6374 (*SPL13*) reverse | GCCACAAATTATCCCAGCACA |
| TC405272 (*NAC*) forward | CGTGACCTGCTTCTCCAACA |
| TC405272 (*NAC*) reverse | TGGCCGAAGGAAGAAGAGAT |
| TC430604 (*PSK1*) forward | CCAACACTGCTCCTCACCAA |
| TC430604 (*PSK1*) reverse | CCTTGTGCTGCGTGTAGATGT |
| Ta.46083 (*HOX9*) forward | AATGTGACCACTCCAAACCCTC |
| Ta.46083 (*HOX9*) reverse | CGGCAACAATACCAAACGAAT |
| TC427997 (*ARF17*) forward | TGCTTGGATGAGTCGGGATA |
| TC427997 (*ARF17*) reverse | GCAGGACACACTGTTCACGA |
| TC398226 (*TCP*) forward | TTCAAGGAGGAAAGGAAGACATAA |
| TC398226 (*TCP*) reverse | AACATTTGGTTTCCGATCACAA |
| TC371524 (*TIR1*) forward | TGAGATGCTGTCAATCGCCT |
| TC371524 (*TIR1*) reverse | GGAAGTGACTCCACAGGGCA |
| Ta.88261 (*SPX*) forward | ACCAAAGCCGAACCTATCCA |
| Ta.88261 (*SPX*) reverse | TCTTGGCGTTTCGCTACCTG |
| TC389301 (*Pol II*) forward | CCAAGGATGATGAGCCCGTA |
| TC389301 (*Pol II*) reverse | GCATCAAGCCAACCAACAGC |
| Ta.103967 forward | TCGCTTGGATCTGAAACCTG |
| Ta.103967 reverse | CATCCCCTTCCAAAAGTTCTG |
| Ta.42302 forward | CCGACCACAAAGAGGCTCTG |
| Ta.42302 reverse | CGGAACACGTCGGAGTTCAT |
| *actin* (AB181991) forward | AGGTGCCCTGAGGTGCTGTT |
| *actin* (AB181991) reverse | GCCAAAATAGAGCCACCGAT |

# The reverse primer complementary to the poly(T) adapter was provided by the miRcute miRNA cDNA synthesis kit (Tiangen, China).
