# Supplementary material for: Small RNA and Degradome Sequencing Reveal Complex Roles of miRNAs and Their Targets in Developing Wheat Grains
Source: PLoS One. 2015 Oct 1;10(10):e0139658. doi: 10.1371/journal.pone.0139658 (PMC4591353; doi:10.1371/journal.pone.0139658)
Supplement: S2 Table — (DOCX) [file pone.0139658.s007.docx]

**S2 Table.** **Distribution of sequence reads in the four small RNA libraries.**

| RNA class | Redundant reads | | | |  | Unique reads | | | |
| --- | --- | --- | --- | --- | --- | --- | --- | --- | --- |
|  | 7 DPA | 14 DPA | 21 DPA | 28 DPA |  | 7 DPA | 14 DPA | 21 DPA | 28 DPA |
| Total clean reads^a^ | 17,133,120 | 14,988,782 | 30,638,998 | 10,090,349 |  | 2,706,941 | 3,367,889 | 2,429,108 | 890,644 |
| Matching EST/Unigene^b^ | 5,378,231 | 5,328,512 | 10,306,959 | 4,123,925 |  | 549,509 | 663,371 | 558,695 | 240,652 |
| Known miRNA^c^ | 621,366 | 266,890 | 361,844 | 164,397 |  | 629 | 705 | 671 | 602 |
| rRNA | 704,390 | 957,007 | 2,200,767 | 749,444 |  | 9,019 | 12,415 | 12,009 | 7,277 |
| tRNA | 72,052 | 105,441 | 208,669 | 87,671 |  | 1,176 | 1,688 | 1,437 | 1,025 |
| snRNA/snoRNA | 15,802 | 20,734 | 35,605 | 17,013 |  | 341 | 445 | 417 | 298 |
| Others^c^ | 15,719,510 | 13,638,710 | 27,832,113 | 9,071,824 |  | 2,695,776 | 3,352,636 | 2,414,574 | 881,442 |

^a^ 18 nt to 28 nt in length.

^b^ Matching NCBI wheat EST database and DFCI Wheat Gene Index.

^c^ Contains all of the unclassified sequences that possibly include new miRNAs.
