# Supplementary material for: Small RNA and Degradome Sequencing Reveal Complex Roles of miRNAs and Their Targets in Developing Wheat Grains
Source: PLoS One. 2015 Oct 1;10(10):e0139658. doi: 10.1371/journal.pone.0139658 (PMC4591353; doi:10.1371/journal.pone.0139658)
Supplement: S3 Table — (DOCX) [file pone.0139658.s008.docx]

**S3 Table. Known miRNAs in the miRBase were identified in our wheat small RNA libraries.**

| **miRNA ID** | **Len**  **(nt)** | **Sequence (5′–3′)** | **TPM^‡^** | | | | **miR*^║^** | **Homology**  **in miRbase^¶^** | **Precursor^†^**  **(EST No.)** |
| --- | --- | --- | --- | --- | --- | --- | --- | --- | --- |
|  |  |  | **7DPA** | **14DPA** | **21DPA** | **28DPA** |  |  |  |
| tae-miR156a^¥^ | 20 | UGACAGAAGAGAGUGAGCAC | 15.99 | 71.99 | 16.35 | 25.77 | Y | tae-miR156 | A |
| tae-miR156b | 21 | UUGACAGAAGAGAGUGAGCAC | 21.65 | 61.25 | 14.49 | 14.96 |  | gma-miR156k(0) | NA |
| tae-miR159a,b | 21 | UUUGGAUUGAAGGGAGCUCUG | 76.64 | 119.22 | 35.22 | 106.44 | a(Y) | tae-miR159a,b | A |
| tae-miR159c | 20 | CUUGGAUUGAAGGGAGCUCU | 4.55 | 30.42 | 2.12 | 4.56 |  | osa-miR159f(0) | NA |
| tae-miR160  tae-miR164 | 21  21 | UGCCUGGCUCCCUGUAUGCCA  UGGAGAAGCAGGGCACGUGCA | 3.85 | 8.87 | 2.84 | 2.77 | N  Y | tae-miR160  tae-miR164 | A  A |
|  |  |  | 22.88 | 53.44 | 23.21 | 25.97 |  |  |  |
| tae-miR165 | 21 | UCGGACCAGGCUUCAUCCCCC | 3.27 | 1.87 | 1.50 | 1.09 |  | ath-miR165a(0) | NA |
| tae-miR166a | 21 | UCGGACCAGGCUUCAUUCCCC | 11845.42 | 3717.98 | 2657.30 | 3347.36 | Y | bdi-miR166a(0) | JV847609 |
| tae-miR166b | 21 | UCGGACCAGGCUUCAUUCCCC | 11845.42 | 3717.98 | 2657.30 | 3347.36 | Y | bdi-miR166a(0) | JV909329 |
| tae-miR166c | 21 | UCGGACCAGGCUUCAAUCCCU | 454.73 | 140.51 | 24.35 | 47.57 |  | osa-miR166k(0) | NA |
| tae-miR166d | 21 | UCGUACCAGGCUUCAUUCCCC | 209.24 | 71.99 | 0.10 | 105.55 |  | bdi-miR166a(1) | NA |
| tae-miR166e | 21 | UCGGACCAGGCUUCAUUCCCU | 23.35 | 10.34 | 4.99 | 3.87 |  | osa-miR166m(0) | NA |
| tae-miR166f | 21 | UCGGACCAGGCUUCAUUCCUU | 13.02 | 6.47 | 0.78 | 2.18 |  | ptc-miR166n(0) | NA |
| tae-miR166g | 21 | UCGAACCAGGCUUCAUUCCCC | 10.51 | 2.74 | 0.03 | 8.32 |  | osa-miR166e(0) | NA |
| tae-miR166h | 21 | UCGGACCAGGCUUCAUUCCUC | 1.23 | 0.60 | 0.46 | 0.40 |  | osa-miR166g(0) | NA |
| tae-miR166i | 21 | UGGGACCAGGCUUCAUUCCCC | 13.37 | 4.94 | 0.62 | 1.59 |  | bdi-miR166a(1) | NA |
| tae-miR166j | 21 | UCGGACCAGGCUUCAUUCCGC | 8.46 | 2.13 | 2.48 | 0.99 |  | bdi-miR166a(1) | NA |
| tae-miR166k | 21 | UCGGACCAGGCUUCAUUCCCA | 7.06 | 1.00 | 2.35 | 2.28 |  | bdi-miR166a(1) | NA |
| tae-miR166l | 21 | UCGGACCAGGCUUCAUUCCAC | 6.89 | 2.13 | 0.62 | 13.08 |  | bdi-miR166a(1) | NA |
| tae-miR167a | 21 | UGAAGCUGCCAGCAUGAUCUA | 5.02 | 9.87 | 8.49 | 23.98 | N | tae-miR167a | A |
| tae-miR167c | 22 | UGAAGCUGCCAGCAUGAUCUGC | 107.34 | 60.71 | 27.09 | 28.74 | Y | tae-miR167c | A |
| tae-miR167d | 22 | UGAAGCUGCCAGCAUGAUCUGA | 85.80 | 305.56 | 88.42 | 190.88 | Y | bdi-miR167c(0) | AEOM01105062 |
| tae-miR167e | 22 | UGAAGCUGCCAGCAUGAUCUGU | 0.18 | 1.53 | 0.39 | 3.27 |  | [ath-miR167d(1)](http://www.mirbase.org/cgi-bin/mirna_entry.pl?acc=MIMAT0000905) | NA |
| tae-miR167f | 22 | UGAAGCUGCCAGCAUGAUCUGG | 0.82 | 0.20 | 0.98 | 1.78 |  | [ath-miR167d(0)](http://www.mirbase.org/cgi-bin/mirna_entry.pl?acc=MIMAT0000905) | NA |
| tae-miR168 | 21 | UCGCUUGGUGCAGAUCGGGAC | 82.82 | 298.49 | 38.45 | 37.66 |  | bdi-miR168(0) | NA |
| tae-miR169a | 21 | CAGCCAAGGAUGACUUGCCGA | 1.05 | 0.20 | 0.00 | 0.79 | Y | osa-miR169a(0) | GAEF01083997 |
| tae-miR169b-5p | 21 | CAGCCAAGGAUGACUUGCCGG | 3.85 | 0.40 | 0.39 | 2.08 |  | osa-miR169b(0) | JP941264 |
| tae-miR169b-3p | 21 | CAAGUUGUCCUUGGCUACACC | 446.97 | 10.27 | 7.83 | 4.66 |  |  | JP941264 |
| tae-miR169c | 21 | UAGCCAAGGAUGAUUUGCCUG | 0.29 | 0.00 | 0.16 | 0.00 | N | bdi-miR169k(0) | JV818128 |
| tae-miR171a | 21 | UGAUUGAGCCGUGCCAAUAUC | 19.26 | 5.40 | 2.61 | 3.96 | Y | tae-miR171a | A |
| tae-miR171b | 21 | UUGAGCCGUGCCAAUAUCACG | 1.58 | 2.20 | 1.24 | 1.59 | N | tae-miR171b | A |
| tae-miR171c | 21 | UGAUUGAGCCGCGCCAAUAUC | 16.58 | 0.93 | 0.46 | 0.00 |  | osa-miR171a(0) | NA |
| tae-miR171d | 21 | UGAGCCGAACCAAUAUCACUC | 1.05 | 3.60 | 2.68 | 1.39 |  | mdm-miR171i(0) | NA |
| tae-miR172a | 21 | AGAAUCUUGAUGAUGCUGCAU | 0.58 | 0.73 | 0.20 | 0.00 |  | osa-miR172a(0) | NA |
| tae-miR172b | 21 | GGAAUCUUGAUGAUGCUGCAU | 0.47 | 0.27 | 0.03 | 0.00 | N | osa-miR172b(0) | AEOM01013727 |
| tae-miR319a | 21 | UUGGACUGAAGGGAGCUCCCU | 1.28 | 7.27 | 5.97 | 7.43 | N | tae-miR319 | A |
| tae-miR319b | 21 | UUGGACUGAAGGGUGCUCCCU | 240.00 | 469.22 | 417.47 | 258.37 |  | tae-miR319(1) | NA |
| tae-miR390 | 21 | AAGCUCAGGAGGGAUAGCGCC | 1.58 | 2.40 | 1.34 | 2.97 | Y | ath-miR390a(0) | AEOM01223153 |
| tae-miR393 | 21 | UCCAAAGGGAUCGCAUUGAUC | 57.96 | 16.81 | 2.35 | 1.49 |  | osa-miR393a(0) | JW008457 |
| tae-miR394 | 20 | UUGGCAUUCUGUCCACCUCC | 4.09 | 3.94 | 1.96 | 3.57 |  | osa-miR394(0) | NA |
| tae-miR395a^¥^ | 20 | UGAAGUGUUUGGGGGAACUC | 0.00 | 0.00 | 0.42 | 0.00 | N | tae-miR395a | A |
| tae-miR395b-d | 20 | UGAAGUGUUUGGGGGAACUC | 0.00 | 0.00 | 0.42 | 0.00 | d(Y) | tae-miR395b(0) | CK194045 |
| tae-miR396a | 21 | UCCACAGGCUUUCUUGAACUG | 2452.79 | 1155.66 | 321.91 | 275.02 | N | tae-miR396 | A |
| tae-miR396b | 21 | UUCCACAGCUUUCUUGAACUG | 816.61 | 201.88 | 83.36 | 75.72 |  | sbi-miR396a(0) | NA |
| tae-miR396c | 21 | UUCCACAGCUUUCUUGAACUU | 40.27 | 12.41 | 0.46 | 0.00 |  | sbi-miR396c(0) | NA |
| tae-miR396d | 21 | UCCACAGGCUUUCUUGAACGG | 10.80 | 0.47 | 2.32 | 1.19 |  | [osa-miR396g(0)](http://www.mirbase.org/cgi-bin/mirna_entry.pl?acc=MIMAT0013833) | NA |
| tae-miR396e | 21 | UCCACAGGCUUUCUUGAACUU | 6.48 | 1.20 | 1.27 | 4.06 |  | osa-miR396e(1) | NA |
| tae-miR396f | 21 | UUCCACAGCUUUCUUGAACGG | 2.80 | 0.40 | 0.75 | 0.40 |  | osa-miR396a(1) | NA |
| tae-miR397 | 21 | UUGAGUGCAGCGUUGAUGAAC | 7.00 | 10.47 | 8.22 | 8.52 | Y | tae-miR397 | A |
| tae-miR398 | 21 | UGUGUUCUCAGGUCGCCCCCG | 0.12 | 0.53 | 0.13 | 0.00 | Y | tae-miR398 | A |
| tae-miR399^¥^ | 21 | UGCCAAAGGAGAAUUGCCCUG | 2.28 | 7.27 | 1.04 | 1.09 | Y | tae-miR399 | A |
| tae-miR408^¥^ | 20 | UGCACUGCCUCUUCCCUGGC | 0.58 | 2.80 | 1.86 | 42.61 | N | tae-miR408 | A |
| tae-miR444c.1 | 21 | UGUUGUCUCAAGCUUGCUGCC | 45.58 | 40.43 | 13.35 | 18.93 | Y | osa-miR444c.1(0) | HX070184 |
| tae-miR444c.2 | 21 | UGCAGUUGUUGUCUCAAGCUU | 1.75 | 0.60 | 0.00 | 0.69 | Y | osa-miR444c.2(0) | HX070184 |
| tae-miR444d | 21 | UGCAGUUGCUGCCUCAAGCUU | 1.63 | 1.13 | 0.13 | 0.00 | N | osa-miR444d.2(0) | CK200584 |
| tae-miR444e | 21 | UGCAGUUGCUGUCUCAAGCUU | 3.27 | 3.40 | 0.59 | 0.00 | N | Hvu-miR444b(0) | BJ233977 |
| tae-miR528 | 21 | UGGAAGGGGCAUGCAGAGGAG | 0.00 | 0.67 | 0.26 | 0.10 |  | osa-miR528(0) | NA |
| tae-miR530 | 21 | UGCAUUUGCACCUGCACCUAC | 0.12 | 0.07 | 0.00 | 0.00 |  | bdi-miR530a(0) | NA |
| tae-miR827 | 21 | UUAGAUGACCAUCAGCAAACA | 25.27 | 68.65 | 64.26 | 43.11 |  | bdi-miR827(0) | NA |
| tae-miR894a | 21 | CGUUUCACGUCGGGUUCACCA | 9.22 | 127.23 | 29.02 | 20.51 |  | ppt- miR894(1) | NA |
| tae-miR894b | 21 | CGAUUCACGUCGGGUUCACCA | 12.43 | 16.61 | 64.56 | 102.87 |  | ppt- miR894(2) | NA |
| tae-miR1117 | 24 | UAGUACCGGUUCGUGGCACGAACC | 0.29 | 0.80 | 0.26 | 0.20 | N | tae-miR1117 | A |
| tae-miR1118 | 23 | CACUACAUUAUGGAAUGGAGGGA | 0.00 | 0.07 | 0.00 | 0.00 | N | tae-miR1118 | A |
| tae-miR1119 | 24 | UGGCACGGCGUGAUGCUGAGUCAG | 0.00 | 0.00 | 0.29 | 0.00 | N | tae-miR1119 | A |
| tae-miR1120b | 21 | UUCUUAUAUUGUGGGACAGAG | 0.18 | 0.00 | 0.16 | 0.00 | N | tae-miR1120b | A |
| tae-miR1120c | 21 | UAAUAUAAGAACGUUUUUGAC | 0.12 | 0.20 | 0.55 | 0.99 | Y | tae-miR1120c | A |
| tae-miR1121 | 22 | AGUAGUGAUCUAAACGCUCUUA | 1.75 | 0.73 | 0.16 | 1.09 | N | tae-miR1121 | A |
| tae-miR1125 | 24 | AACCAACGAGACCAACUGCGGCGG | 0.00 | 0.20 | 0.00 | 0.00 | N | tae-miR1125 | A |
| tae-miR1127b | 21 | ACAAGUAUUUCUGGACGGAGG | 0.23 | 0.13 | 0.55 | 0.69 | N | tae-miR1127b | A |
| tae-miR1128 | 21 | UACUACUCCCUCCGUCCGAAA | 0.00 | 0.07 | 0.00 | 0.00 | N | tae-miR1128 | A |
| tae-miR1130a | 23 | CCUCCGUCUCGUAAUGUAAGACG | 0.12 | 0.00 | 0.00 | 0.00 | N | tae-miR1130a | A |
| tae-miR1130b | 21 | UCUUAUAUUAUGGGACGGAGG | 1.11 | 0.87 | 0.33 | 0.00 | N | tae-miR1130b | A |
| tae-miR1133 | 22 | CAUAUACUCCCUCCGUCCGAAA | 0.06 | 0.00 | 0.16 | 0.40 | N | tae-miR1133 | A |
| tae-miR1135 | 24 | CUGCGACAAGUAAUUCCGAACGGA | 0.82 | 5.07 | 5.03 | 2.38 | Y | tae-miR1135 | A |
| tae-miR1136 | 24 | UUGUCGCAGGUAUGGAUGUAUCUA | 1.75 | 5.4 | 7.41 | 10.7 | Y | tae-miR1136 | A |
| tae-miR1137a | 20 | UAGUACAAAGUUGAGUCAUC | 0.00 | 0.20 | 0.00 | 0.00 | N | tae-miR1137a | A |
| tae-miR1137b | 21 | UCCGUUCCAGAAUAGAUGACC | 0.82 | 0.80 | 0.00 | 0.00 | N | tae-miR1137b | A |
| tae-miR1432a | 21 | AUCAGGAGAGAUGACACCGAC | 2.33 | 1.20 | 0.00 | 0.00 |  | osa-miR1432(0) | NA |
| tae-miR1432b | 21 | CUCAGGAGAGAUGACACCGAC | 0.18 | 0.27 | 0.00 | 0.00 |  | sbi-miR1432(0) | NA |
| tae-miR1436 | 21 | AUAUUAUGGGGCGGAGGGAGU | 0.06 | 0.27 | 0.23 | 0.00 | N | osa-miR1436(2) | BJ270174 |
| tae-miR1847 | 21 | ACCUGCAGUUGGGCCAAUGAC | 0.18 | 1.80 | 1.11 | 0.00 | Y | tae-miR1847 | A |
| tae-miR1878 | 24 | AUUUGUAGUGUUCGGAUUGAGUUU | 16.11 | 27.62 | 9.76 | 26.56 |  | bdi-miR1878(1) | NA |
| tae-miR5048 | 22 | UUUGCAGGUUUUAGGUCUAAGU | 141.25 | 139.50 | 261.24 | 328.83 | N | tae-miR5048 | A |
| tae-miR5049a | 21 | AAUAUGGAUCGGAGGGAGUAC | 0.06 | 0.33 | 0.13 | 0.00 | N | tae-miR5049 | A |
| tae-miR5049b | 21 | AAUUAAUAUGGAUCGGAGGGA | 0.29 | 0.60 | 0.07 | 0.30 | Y | hvu-miR5049f(0) | HX189484 |
| tae-miR5054 | 18 | UCCCCACGGUCGGCGCCA | 3.04 | 6.87 | 2.97 | 1.59 |  | bdi-miR5054(0) | NA |
| tae-miR5062a | 23 | UGAACCUUAGGGAACAGCCGCAU | 3.33 | 3.27 | 0.13 | 0.00 | Y | tae-miR5062 | A |
| tae-miR5062b | 23 | UGAACCUUGGGGAAAAGCCGCAU | 22.53 | 22.22 | 9.73 | 30.92 |  | bdi-miR5062a(1) | NA |
| tae-miR5064a | 21 | UGAAUUUGUCCAUAGCAUCAG | 30.41 | 32.69 | 14.23 | 10.01 |  | bdi-miR5064a(1) | NA |
| tae-miR5064b | 21 | UGAAUUUGUCCAUAGCAUCAU | 1.34 | 1.27 | 0.62 | 0.69 |  | bdi-miR5064a(2) | NA |
| tae-miR5071a | 21 | UCAAGCAUCAUAUCAUGGACA | 57.61 | 101.28 | 30.91 | 36.27 |  | osa-miR5071(1) | HX151716 |
| tae-miR5071b | 21 | UCAAGCAUCAUAUCAUGGACU | 7.70 | 7.61 | 21.31 | 49.16 |  | osa-miR5071(2) | NA |
| tae-miR5071c | 21 | UCAAGCAUCAUGUCAUGGACA | 8.17 | 4.34 | 4.50 | 11.99 |  | osa-miR5071(2) | NA |
| tae-miR5083 | 21 | CAGACUACAAUUAUCUGAUCA | 9.63 | 0.00 | 1.89 | 1.88 |  | osa-miR5083(1) | NA |
| tae-miR5084 | 24 | AUACAGUACUGCAGAGGAUCCUAA | 0.06 | 1.00 | 0.00 | 1.19 | N | tae-miR5084 | A |
| tae-miR5139 | 18 | AACCUGGCUCUGAUACCA | 34.09 | 51.71 | 10.09 | 30.62 |  | rgl-miR5139(1) | NA |
| tae-miR5175 | 21 | UUCCAAAUUACUCGUCGUGGU | 30.18 | 64.85 | 14.03 | 10.21 | N | tae-miR5175 | A |
| tae-miR5384 | 21 | UGAGCGCGCCGCCGUCGAAUG | 0.41 | 0.40 | 0.65 | 0.00 | Y | tae-miR5384 | A |
| tae-miR6197 | 21 | UCUGUAAACAAAUGUAGGACG | 0.93 | 1.27 | 0.72 | 0.00 | N | tae-miR6197 | A |
| tae-miR6300 | 18 | GUCGUUGUAGUAUAGUGG | 6.89 | 1.73 | 3.69 | 11.60 |  | gma-miR6300(0) | NA |
| tae-miR6478a | 20 | CCGACCUUAGCUCAGUUGGU | 42.14 | 77.39 | 22.59 | 7.73 |  | ptc-miR6478(0) | NA |
| tae-miR6478b | 20 | CCGACUUUAGCUCAGUUGGU | 50.43 | 61.18 | 79.64 | 37.86 |  | ptc-miR6478(1) | NA |
| tae-miR9652 | 22 | CCUGUUUGUCAUUAAGUUUCUU | 5.02 | 8.47 | 7.67 | 3.07 | N | tae-miR9652 | A |
| tae-miR9653a-3p | 21 | UUUGAGACUUUGGCCAUGGCC | 185.08 | 447.13 | 93.51 | 39.05 | Y | tae-miR9653a | A |
| tae-miR9653b^¥^ | 23 | CGUGGCCAAGGUCUCUUGAGGCU | 155.72 | 169.86 | 434.35 | 65.41 | Y | tae-miR9653b | A |
| tae-miR9654a | 22 | UUCUGAAAGGCUUGAAGCGAAU | 0.12 | 0.33 | 0.00 | 0.00 | Y | tae-miR9654a | A |
| tae-miR9654b | 22 | UUCCGAAAGGCUUGAAGCGAAU | 4.32 | 2.47 | 0.59 | 0.00 | Y | tae-miR9654b | A |
| tae-miR9655-5p | 22 | UCGGCUACUUCCUUUCCCUUGC | 127.59 | 85.13 | 67.23 | 88.00 |  | tae-miR9655 | A |
| tae-miR9655-3p | 21 | CAAGGGAAGGAAGUAGCCAAC | 34.20 | 36.76 | 5.45 | 3.27 |  | tae-miR9655 | A |
| tae-miR9656 | 21 | CUUCGAGACUCUGAACAGCGG | 1.40 | 1.33 | 1.80 | 2.18 | Y | tae-miR9656 | A |
| tae-miR9657b | 21 | UUCGUCGGAGAAGCAUGUUGC | 0.23 | 0.20 | 0.16 | 1.09 | N | tae-miR9657b | A |
| tae-miR9658 | 21 | AUCGUUCUGGGUGAAUAGGCC | 25.51 | 21.68 | 5.65 | 4.26 | Y | tae-miR9658 | A |
| tae-miR9659-5p | 21 | UGCUGGGAAAACUAUUGAACU | 35.19 | 3.34 | 1.93 | 0.00 |  | tae-miR9659 | A |
| tae-miR9659-3p | 22 | UCCAAUGGUUGUUCACGGCAUC | 45.29 | 24.42 | 3.52 | 6.34 |  | tae-miR9659 | A |
| tae-miR9660 | 20 | UUGCGAGCAACGGAUGAAUC | 3.33 | 5.07 | 1.96 | 0.69 | Y | tae-miR9660 | A |
| tae-miR9661 | 21 | UGAAGUAGAGCAGGGACCUCA | 0.70 | 0.20 | 0.00 | 0.00 | N | tae-miR9661 | A |
| tae-miR9662a-5p | 21 | GCGGCUCUGUGGUGUUCAAGC | 295.51 | 79.73 | 269.59 | 153.81 |  | tae-miR9662a | A |
| tae-miR9662a-3p | 21 | UUGAACAUCCCAGAGCCACCG | 158.82 | 186.87 | 37.76 | 68.28 |  | tae-miR9662a | A |
| tae-miR9662b-5p | 21 | GGCGGCUCUCUGGUGUUCAAG | 26.67 | 8.74 | 6.36 | 4.06 |  | tae-miR9662b | A |
| tae-miR9662b-3p | 21 | UGAACAUCCCAGAGCCACCGG | 43.60 | 24.62 | 21.67 | 128.24 |  | tae-miR9662b | A |
| tae-miR9663 | 21 | AAGCGUAGUCGAACGAAUCUG | 46.28 | 36.29 | 26.31 | 40.93 | Y | tae-miR9663 | A |
| tae-miR9664 | 21 | UUGCAGUCCUCGAUGUCGUAG | 2.04 | 1.53 | 1.63 | 3.77 | Y | tae-miR9664 | A |
| tae-miR9665 | 23 | GCUAGCAGUGUAAACUCAAAUCA | 2.98 | 1.07 | 0.29 | 0.00 | Y | tae-miR9665 | A |
| tae-miR9666a | 22 | CGGUAGGGCUGUAUGAUGGCGA | 3.44 | 136.64 | 235.16 | 239.93 | Y | tae-miR9666a | A |
| tae-miR9666b | 22 | CGGUUGGGCUGUAUGAUGGCGA | 3.79 | 53.71 | 103.30 | 73.14 | Y | tae-miR9666b | A |
| tae-miR9668 | 21 | CCAAUGACAAGUAUUUUCGGA | 0.35 | 0.00 | 0.10 | 0.00 | Y | tae-miR9668 | A |
| tae-miR9669^¥^ | 22 | UACUGUGGGCACUUAUUUGACA | 232.30 | 239.45 | 100.66 | 90.68 | Y | tae-miR9669 | A |
| tae-miR9670-5p | 21 | UUCUUCAAGUACUCCACUUUU | 43.60 | 65.92 | 16.87 | 9.41 |  | tae-miR9670 | A |
| tae-miR9670-3p | 21 | AGGUGGAAUACUUGAAGAAGA | 0.82 | 1.20 | 0.16 | 0.59 |  | tae-miR9670 | A |
| tae-miR9671 | 22 | UGACUUUACACAACUGUCCGGC | 0.35 | 0.13 | 0.52 | 0.00 | N | tae-miR9671 | A |
| tae-miR9672a | 21 | CCACGACUGUCAUUAAGCAUC | 0.58 | 0.93 | 0.65 | 1.49 | N | tae-miR9672a | A |
| tae-miR9672b | 21 | UACCACGACUGUCAUUAAGCA | 40.91 | 85.26 | 7.12 | 7.53 | Y | tae-miR9672b | A |
| tae-miR9673 | 20 | UAAGAAGCAAAUAGCACAUG | 0.53 | 0.20 | 0.33 | 0.00 | Y | tae-miR9673 | A |
| tae-miR9674a^¥^ | 21 | AUAGCAUCAUCCAUCCUACCA | 24.75 | 37.49 | 12.63 | 20.71 | Y | tae-miR9674a | A |
| tae-miR9674b | 21 | AUAGCAUCAUCCAUCCUACCC | 149.07 | 164.12 | 105.36 | 214.46 | Y | tae-miR9674b | A |
| tae-miR9675 | 21 | UUUAUGAUCACUCUCGUUUUG | 2.57 | 3.27 | 5.25 | 8.13 | N | tae-miR9675 | A |
| tae-miR9676 | 22 | UGGAUGUCAUCGUGGCCGUACA | 0.82 | 1.80 | 0.13 | 0.00 | Y | tae-miR9676 | A |
| tae-miR9677a | 22 | UGGCCGUUGGUAGAGUAGGAGA | 30.12 | 0.80 | 0.00 | 0.00 | Y | tae-miR9677a | A |
| tae-miR9678 | 22 | UCUGGCGAGGGACAUACACUGU | 0.00 | 14.74 | 30.71 | 195.53 | Y | tae-miR9678 | A |
| tae-miR9679 | 21 | CAGAACCAGAAUGAGUAGCUC | 31.75 | 26.15 | 12.24 | 25.47 | N | tae-miR9679 | A |
| tae-miR9772 | 21 | UGAGAUGAGAUUACCCCAUAC | 18.56 | 31.89 | 3.52 | 5.85 | Y | tae-miR9772 | A |
| tae-miR9773 | 24 | UUUGUUUUUAUGUUAUUUUGUGAA | 21.13 | 17.61 | 6.01 | 0.69 | N | tae-miR9773 | A |
| tae-miR9774^¥^ | 23 | AACAAGAUAUUGGGUAUUUCUGU | 9.11 | 11.68 | 20.07 | 0.79 | N | tae-miR9774 | A |
| tae-miR9776 | 21 | UGGACGAGGAUGUGCAACUGC | 70.57 | 92.27 | 15.54 | 18.83 | N | tae-miR9776 | A |

‡ TPM: transcripts per million. The miRNA abundance was counted according to the reads of defined miRNAs and their ± 2 nt variants on the precursors.

║ Y: miRNA* species (or ± 1 nt variants) for their corresponding miRNAs were sequenced in our small RNA libraries. For miRNAs with multiple members, only the members with sequenced miRNA* are listed and Y shown in parenthesis. N: miRNA* unsequenced.

¶ The most homologous plant miRNAs in the miRbase were listed with mismatches shown in parenthesis.

† A: available in miRbase; NA: not available.

¥ If a variant has far more sequence reads than the reported miRNA, this variant is in place of the reported one and its sequence and length are shown in blue.
