# Supplementary material for: Small RNA and Degradome Sequencing Reveal Complex Roles of miRNAs and Their Targets in Developing Wheat Grains
Source: PLoS One. 2015 Oct 1;10(10):e0139658. doi: 10.1371/journal.pone.0139658 (PMC4591353; doi:10.1371/journal.pone.0139658)
Supplement: S4 Table — (DOCX) [file pone.0139658.s009.docx]

**S4 Table. Wheat miRNAs not annotated in the miRbase but reported elsewhere were identified in our libraries.**

| **miRNA ID** | **Len**  **(nt)** | **Sequence (5′–3′)** | **TPM^‡^** | | | | **miR*^║^** | **Precursor**  **(EST No.)** | **Reference** |
| --- | --- | --- | --- | --- | --- | --- | --- | --- | --- |
|  |  |  | **7DPA** | **14DPA** | **21DPA** | **28DPA** |  |  |  |
| tae-miR2008 | 19 | GACUCCGUGGCCCAAUGGA | 52.00 | 93.27 | 33.26 | 22.50 | N | CL900687 | [1] |
| tae-miR2009a | 22 | UGAGAAGGUAGAUCAUAAUAGC | 4684.26 | 4018.47 | 2891.54 | 5824.97 | Y | CK193889 | [1] |
| tae-miR2009b | 22 | UUAGAUGAGAAGGCAGAUCAUA | 11.85 | 10.34 | 9.76 | 21.21 | Y | DR736484 | [1] |
| tae-miR2009c | 22 | UCAGAUGAGAAGGCAGAUCAUA | 18.50 | 8.67 | 7.64 | 20.42 | Y | CK193889 | [1] |
| tae-miR2018 | 20 | GCCCGUCUAGCUCAGUUGGU | 12.26 | 11.74 | 8.78 | 5.85 | N | BE405505 | [1] |
| tae-miR2023a^¥^ | 19 | UUUUGCCGGUUGAACGACC | 4.73 | 1.40 | 0.91 | 1.09 | N | HP624615 | [1] |
| tae-miR2023b^¥^ | 19 | UUUUGCUGGUUGAACGACC | 0.23 | 0.13 | 0.00 | 0.00 | N | HX096644 | [1] |
| Ta-miR2004 | 21 | CAUCUAUUUUGGAACGGAGGG | 6.48 | 3.34 | 3.10 | 1.98 | N | CJ585232 | [2] |
| Ta-miR2015 | 21 | UCUUAUAUUGUGGGACGGAGG | 0.76 | 0.00 | 0.00 | 0.50 | N | CJ684152 | [2] |
| Ta-miR023b | 22 | UCGCAAAUAAUGGUGGCCCUCG | 1.93 | 3 | 0.95 | 2.28 | Y | BQ607248 | [3] |
| Ta-miR033^¥^ | 21 | UCAAAGGAUGAGCAAAUACUG | 2.33 | 1.73 | 5.19 | 8.92 | N | CA650420 | [3] |
| Ta-miR068 | 20 | CUCUCUCGGGAGGGCUGAUC | 1.05 | 3.4 | 0 | 0 | N | CJ622557 | [3] |
| Ta-miR106 | 21 | CGGUGGAGCUGGUUGAUGGAC | 1.4 | 0.6 | 0.46 | 0 | N | CK209252 | [3] |
| Ta-miR128 | 23 | GUGGAUGAUGAGAUCACAAGUAA | 1.93 | 10.67 | 16.45 | 2.38 | N | CJ723759 | [3] |
| Ta-miR132 | 21 | UAUAAACUUGGUCAAAGUUUG | 0.47 | 0.2 | 0.1 | 0 | N | BE443030 | [3] |
| Ta-miR154-5p | 22 | GGCGAGGGACAUACACUGUACA | 0 | 4.67 | 10.71 | 4.96 |  | CJ544247 | [3] |
| Ta-miR154-3p | 21 | UACAGUUUAUGUCCCCGGCAG | 0 | 0.53 | 1.66 | 2.58 |  | CJ544247 | [3] |
| Ta-miR158 | 21 | AAGACAACUAAUUUGGGACGG | 0.35 | 0.33 | 0.55 | 1.78 | N | CJ777288 | [3] |
| tae-miR3009a | 21 | UGGUCUGUGUUUGUUUCAAAC | 0.47 | 4.07 | 0.20 | 1.59 | N |  | [4] |
| tae-miR3013a | 21 | UGUUGCAUGACAAGUUGAGCA | 5.60 | 2.40 | 1.70 | 0 | Y |  | [4] |
| tae-miR3032a | 21 | UUAAGAACAGCAGGGCAUUUU | 0.18 | 0 | 0 | 0 | Y |  | [4] |
| tae-miR3065a | 21 | UUCGCCGGAGCAGCGUGCAGA | 0.23 | 0 | 0.26 | 0 | Y |  | [4] |
| tae-miR3074a | 21 | UUGAGACGAACACAGACCAAC | 2.98 | 7.94 | 1.37 | 3.67 | Y |  | [4] |
| tae-miR3081a | 21 | UAAAGCGUAGUCGAACGAAUC | 1.87 | 2.34 | 2.22 | 1.78 | Y |  | [4] |
| tae-miR3082a | 20 | UAAGAAGCAAAUAGCACAUG | 0.53 | 0.20 | 0.33 | 0 | Y |  | [4] |
| tae-miR3084a | 22 | UAAUCUUCUGGAUAUAUGCUUA | 1.58 | 0.47 | 0 | 1.09 | Y |  | [4] |
| tae-miR3086a | 21 | UACGGCCUGAUGACAUCCACA | 3.79 | 4.20 | 1.14 | 0.40 | Y |  | [4] |
| tae-miR3086b | 21 | UACGGCCUGAUGACAUCCACG | 1.23 | 0 | 1.08 | 0 | N |  | [4] |
| tae-miR3089a | 21 | UAGAAUGGCUGGUGCUAUGGA | 1.75 | 4.07 | 1.96 | 6.94 | Y |  | [4] |
| tae-miR3092a | 21 | UAUCUGGACAAAUCUGAGACA | 0.41 | 0.20 | 1.11 | 0.30 | N |  | [4] |
| tae-miR3094a | 21 | UAUUAGUUGUCGCUGAAACGG | 0.29 | 0.00 | 0.62 | 0 | N |  | [4] |
| tae-miR3098a | 21 | UCAUCUGGCAUUGCUUUCUCU | 0.41 | 0.73 | 0.62 | 0 | Y |  | [4] |
| tae-miR3101a | 21 | UCGCAAAUAAUGGUGGCUCUC | 0.41 | 2.54 | 0.26 | 0 | Y |  | [4] |
| tae-miR3105a | 21 | UCUGAUUUACUCGUCGUGGUU | 1.23 | 0.87 | 0.29 | 1.09 | Y |  | [4] |
| tae-miR3110a | 21 | UCUGUUCACAAAUGUAAGACG | 0.18 | 0 | 0 | 0 | N |  | [4] |
| tae-miR3118a | 21 | UUUGUCUAGAUACGGAUAUAU | 0.18 | 0.47 | 0.13 | 0 | N |  | [4] |
| tae-miR3130a | 22 | UGGAUGUCAUCGUGGCCGUACA | 0.41 | 1.73 | 0 | 0 | Y |  | [4] |
| tae-miR3132a | 22 | UGGGCAAGUCACCCUGGCUACC | 1.23 | 1.60 | 0 | 0 | N |  | [4] |
| tae-miR3134a | 21 | UUGAAUUUGUCCAUAGCAUCA | 3.27 | 3.07 | 0.62 | 2.38 | N |  | [4] |

‡ TPM: transcripts per million. The miRNA abundance was counted according to the reads of defined miRNAs and their ± 2 nt variants on the precursors.

║ Y: miRNA* species (or ± 1 nt variants) for their corresponding miRNAs were sequenced in our small RNA libraries. For miRNAs with multiple members, only the members with sequenced miRNA* are listed and Y shown in parenthesis. N: miRNA* unsequenced.

¥ If a variant has far more sequence reads than the reported miRNA, this variant is in place of the reported one and its sequence and length are shown in blue.

**Reference**

1. Wei B, Cai T, Zhang R, Li A, Huo N, Li S, et al. (2009) Novel microRNAs uncovered by deep sequencing of small RNA transcritomes in bread wheat (*Triticum aestivum* L.) and *Brachypodium distachyon* (L.) Beauv. Funct Integr Genomics 9: 499–511.
2. Xin M, Wang Y, Yao Y, Xie C, Peng H, Ni Z, Sun Q (2010) Diverse set of microRNAs are responsive to powdery mildew infection and heat stress in wheat (*Triticum aestivum* L.). BMC Plant Biol 10: 123.
3. Meng F, Liu H, Wang K, Liu L, Wang S, Zhao Y, et al. (2013) Development-associated microRNAs in grains of wheat (*Triticum aestivum* L.). BMC Plant Biol 13: 140.
4. Sun F, Guo G, Du J, Guo W, Peng H, Ni Z, et al. (2014) Whole-genome discovery of miRNAs and their targets in wheat (*Triticum aestivum* L.). BMC Plant Biol 14: 142.
