# Supplementary material for: Small RNA and Degradome Sequencing Reveal Complex Roles of miRNAs and Their Targets in Developing Wheat Grains
Source: PLoS One. 2015 Oct 1;10(10):e0139658. doi: 10.1371/journal.pone.0139658 (PMC4591353; doi:10.1371/journal.pone.0139658)
Supplement: S5 Table — (DOCX) [file pone.0139658.s010.docx]

**S5 Table. Predicted and verified targets of newly identified miRNAs.**

| **miRNA** | **Predicted targets^#^**  **(Unigene/EST)** | **Putative functions of targets** |
| --- | --- | --- |
|  |  |  |
| miR5048.2 | Ta.100067 (2)  **Ta.90063 (2)**  **Ta.40570 (2)**  **Ta.103967 (2.5)**  **Ta.115136 (2.5)**  Ta.48022 (2.5)  Ta.41454 (2.5)  Ta.10578 (3)  Ta.86927 (3)  Ta.109260 (3)  Ta.166907 (3) | Cysteine-rich receptor-like protein kinase  Cysteine-rich receptor-like protein kinase  Leucine-rich repeat receptor-like serine/threonine-protein kinase  Cysteine-rich receptor-like protein kinase  Cysteine-rich receptor-like protein kinase  Cysteine-rich receptor-like protein kinase  G-type lectin S-receptor-like serine/threonine-protein kinase  Cysteine-rich receptor-like protein kinase  Cysteine-rich receptor-like protein kinase  Cysteine-rich receptor-like protein kinase  Cysteine-rich receptor-like protein kinase |
| Ta-miRn1 | Ta.25217 (1.5)  Ta.57005 (2)  Ta.71280 (2.5)  **Ta.161175 (3)**  **Ta.55347 (3.5)**  **Ta.167568 (3.5)** | Serine/threonine-protein kinase RIO1  Regulator of Vps4 activity in the MVB pathway  Dehydrin  Coiled-coil domain containing protein (DUF2052)  Frigida-like protein  Disease resistance protein RPM1-like |
| Ta-miRn2 | TC390008 (0.5)  **TC406240 (1)**  Ta.50813 (0.5)  Ta.2822 (1)  Ta.13587 (1.5)  Ta.68223 (1.5)  Ta.65886 (1.5) | Aquaporin NIP1-1 like  Sulfite oxidase  Mitochondrial inner membrane protein OXA1-like  Phosphosulfolactate synthase-related protein  Beta-glucosidase 32-like  Antagonist of mitotic exit network protein 1  Serine carboxypeptidase-like 18-like |
| Ta-miRn3 | **TC438261 (1)**  Ta.31823 (1)  TC404523 (1.5)  TC440118 (1.5)  TC378689 (1.5)  **TC447077 (1.5)**  **Ta.91646 (1.5)**  **Ta.203374 (2.5)** | Vacuolar-sorting receptor 4  Ras-related protein RABF1-like  Cell number regulator 2-like  C2H2 zinc finger protein  Delta1-pyrroline-5-carboxylate synthetase  Protein TIFY 10B-like  Beta-adaptin-like protein A-like  Cysteine-rich receptor-like protein kinase 41 |
| Ta-miRn4 | Ta.50769 (0.5)  TC402655 (0.5)  TC377059 (0.5)  TC430693 (0.5)  TC381643 (0.5)  TC439987 (0.5)  TC376672 (0.5)  TC447447 (1) | Adenylosuccinate synthetase 2  Peroxisome biogenesis protein 12  Thiosulfate sulfurtransferase  BEL1-like homeodomain protein 6  DNA-directed RNA polymerase II  Soluble inorganic pyrophosphatase-like  Ribulose bisphosphate carboxylase small chain  Methyltransferase PMT23-like |
| Ta-miRn5 | TC394132 (1)  TC369729 (2) | Hypothetical protein  LRK14 |
| Ta-miRn6 | Ta.78774 (2.5)  Ta.187583 (2.5) | Unknown  Unknown |
| Ta-miRn7 | TC420685 (0.5)  TC407053 (0.5)  TC388450 (2)  CJ883403 (2)  TC452182 (2)  TC381279 (2)  **TC433106 (2)**  **Ta.106901 (1)**  **Ta.76328 (2)** | 3-oxoacyl carrier protein synthase  NADPH-cytochrome P450 reductase  Aminolevulinic acid dehydratase  Cysteine synthase 2-like  Glyoxalase II  Metallo-beta-lactamase-like  Cell division protein ftsY homolog  Unknown  Cell division protein ftsY homolog |
| Ta-miRn8 | **Ta.42302 (1)**  TC390008 (1)  TC435864 (1)  TC374211 (1)  TC379242 (1)  TC399594 (1.5)  TC405876 (2)  TC371157 (2.5)  **Ta.180180 (3)** | MIT (microtubule interacting and transport) domain protein  Aquaporin NIP1-1 like  Small nuclear ribonucleoprotein-like  C2H2 zinc-finger protein  [Acyl-(acyl-carrier-protein) desaturase](http://blast.ncbi.nlm.nih.gov/Blast.cgi#alnHdr_475613074)  Receptor-like protein kinase  E3 ubiquitin-protein ligase  E3 ubiquitin-protein ligase  Unknown |
| Ta-miRn9 | Ta.47541 (1.5)  Ta.52424 (3) | MIKC-type MADS-box transcription factor WM32A/B  MIKC-type MADS-box transcription factor WM30 |
| Ta-miRn10 | TC436708 (0.5)  TC390364 (1.5)  TC403274 (2)  **CJ531707 (2.5)**  TC394690 (3)  TC404007 (3)  TC441900 (3)  TC424561 (3)  TC401095 (3) | [Defensin-like protein](http://blast.ncbi.nlm.nih.gov/Blast.cgi#alnHdr_475549963)  Heavy-metal-associated domain-containing protein  Transcription factor bHLH35-like  Unknown  Monodehydroasorbate reductase  Monodehydroasorbate reductase  Monodehydroasorbate reductase  Monodehydroasorbate reductase  Monodehydroasorbate reductase |
| Ta-miRn11 | − | − |
| Ta-miRn12 | TC398598 (0.5)  TC442941 (0.5)  CK204669 (2)  CK205018 (2) | PolI-like DNA polymerase  PolI-like DNA polymerase  6-phosphogluconolactonase 4  6-phosphogluconolactonase 4 |
| Ta-miRn13 | TC461985 (1)  TC436262 (2.5) | Mitogen-activated protein kinase (MAPK)  Cyclic nucleotide-gated ion channel 20 |
| Ta-miRn14 | TC452716 (0.5)  TC384785 (0.5)  TC403565 (1)  TC378689 (1)  TC38723 (1.5)  TC373825 (2)  TC456944 (2)  TC435003 (2)  TC395015 (2.5)  **CA635704 (2.5)** | Ribosomal protein L11  NADH dehydrogenase 1 beta subcomplex subunit 3-B-like  Ubiquitin-like protein 5-like  Delta1-pyrroline-5-carboxylate synthetase  Zinc transporter ZTP29-like  Ferrtin  Ferrtin  CBS domain-containing protein  [Proline-rich protein](http://blast.ncbi.nlm.nih.gov/Blast.cgi#alnHdr_78707783)  Unknown |
| Ta-miRn15 | TC397212 (0.5) | Eukaryotic peptide chain release factor subunit 1-3 |
| Ta-miRn16 | CK165275 (0.5)  CK164310 (0.5)  TC397832 (0.5)  TC438261 (1)  TC377378 (1)  TC401189 (1.5)  TC405058 (1.5)  TC440118 (2)  TC377852 (2) | Cold acclimation protein WCOR413  Cold acclimation protein WCOR413  Folate-biopterin transporter 4-like  Vacuolar-sorting receptor 4  MtN19-like protein  Uridine 5'-monophosphate synthase  Serine/threonine-protein kinase  C2H2 zinc finger protein  NAC domain-containing protein |
| Ta-miRn17 | TC390615 (1)  TC403565 (1)  TC378689 (1)  TC446650 (1.5)  TC377378 (1.5)  TC440118 (2)  TC372628 (2)  **TC369686 (2)**  **Ta.76242 (2)**  **Ta.33781 (3)** | Homocysteine S-methyltransferase 3  Ubiquitin-like protein 5-like  Delta1-pyrroline-5-carboxylate synthetase  mtN19-like protein  mtN19-like protein  C2H2 zinc finger protein  Chlorophyll a/b-binding protein precursor  WD-40 repeat-containing protein  Mitochondrial-processing peptidase subunit alpha-like  Protein furry homolog-like protein |
| Ta-miRn18 | **Ta.37221 (1)**  TC389889 (0.5)  TC412717 (0.5)  TC403709 (0.5)  TC390819 (1)  TC441321 (1)  TC392608 (1.5)  TC448038 (2)  TC412824 (2)  TC369017 (2) | PsbP family protein  Dof zinc finger protein  Brown planthopper-induced resistance protein 1  Ubiquitin-like-specific protease ESD4  Ras-related protein RABC2a  Serine/threonine-protein phosphatase BSL2-like protein  Glutathione transferase  GRIP-like protein  Defensin-like protein 1  MADS-box transcription factor TaAGL11 |
| Ta-miRn19 | CV766835 (2) | Unknown |
| Ta-miRn20 | − | − |
| Ta-miRn21 | − | − |

# Targets in bold indicated that they were supported by degradome sequencing data.
