# Supplementary material for: Small RNA and Degradome Sequencing Reveal Complex Roles of miRNAs and Their Targets in Developing Wheat Grains
Source: PLoS One. 2015 Oct 1;10(10):e0139658. doi: 10.1371/journal.pone.0139658 (PMC4591353; doi:10.1371/journal.pone.0139658)
Supplement: S6 Table — (DOCX) [file pone.0139658.s011.docx]

**S6 Table. Predicted and verified targets of differentially expressed miRNAs.**

| **miRNA family** | **Predicted targets^#^**  **(Unigene/EST)** | **Putative functions of targets** |
| --- | --- | --- |
|  |  |  |
| miR156 | Ta.29809 (1)  Ta.3711 (2)  **Ta.140644 (1)**  Ta.7021 (2)  **Ta.6374 (2)^a^**  Ta.68761 (1)  Ta.99592 (1)  Ta.101126 (1)  Ta.101595 (1) | Squamosa promoter-binding-like protein 2 (SPL2)-like  Squamosa promoter-binding-like protein 3 (SPL3)-like  Squamosa promoter-binding-like protein 7 (SPL7)-like  Squamosa promoter-binding-like protein 11 (SPL11)-like  Squamosa promoter-binding-like protein 13 (SPL13)-like  Squamosa promoter-binding-like protein 14 (SPL14)-like  Squamosa promoter-binding-like protein 16 (SPL16)-like  Squamosa promoter-binding-like protein 18 (SPL18)-like  Squamosa promoter-binding-like protein 18 (SPL18)-like |
| miR159 | TC368630 (2.5)  TC421314 (3)  TC388353 (2.5) | Transcription factor GAMYB  Transcription factor GAMYB  NB-ARC domain containing protein |
| miR164 | **TC371535 (2)**  **TC376198 (2)**  **TC390810 (2)**  TC394945 (2)  **TC405272 (2)**  TC410195 (2)  TC416811 (2)  TC429623 (2)  Ta.7205 (2.5)  **TC430604 (3)**  **TC378209 (3.5)**  **TC404170 (3.5)** | NAC transcription factor  NAC transcription factor  NAC transcription factor  NAC transcription factor  NAC transcription factor  NAC transcription factor  NAC transcription factor  NAC transcription factor  Mitogen-activated protein kinase  Phytosulfokine-alpha 1 precursor (PSK1)  Phytosulfokine-alpha 1 precursor (PSK1)  Phytosulfokine-alpha 1 precursor (PSK1) |
| miR165/166 | **Ta.47955 (1.5)**  **Ta.46083 (3)**  **TC404537 (3)**  Ta.27428 (3) | Homeobox-leucine zipper protein HOX33-like  Homeobox-leucine zipper protein HOX9-like  Homeobox-leucine zipper protein HOX32-like  MATE efflux family protein |
| miR167 | TC401632 (3)  **TC392555 (4)**  **TC392558 (4)**  **TC427997 (4)**  **Ta.6394 (4)**  Ta.35036 (4) | Auxin response factor 12-like  Auxin response factor 17-like  Auxin response factor 17-like  Auxin response factor 17-like  Auxin response factor 25-like  Auxin response factor 6-like |
| miR168 | **TC379968 (4)**  **TC388344 (4)** | Argonaute 1B (AGO1B)  Argonaute 1B (AGO1B) |
| miR169b-5p | TC401480 (1.5) | Unknown |
| miR171 | **TC398770 (0.5)^a^** | Scarecrow-like transcription factor 1 (SCL1) |
| miR319 | TC368630 (2)  TC421314 (2)  **TC398226 (3)^a^**  **TC432120 (3)** | Transcription factor GAMYB  Transcription factor GAMYB  TCP transcription factor  TCP transcription factor |
| miR393 | CA484228 (1)  **TC422531 (1)**  **TC371524 (1)**  **TC388306 (1)** | Transport inhibitor response 1 (TIR1)-like protein  Transport inhibitor response 1 (TIR1)-like protein  Transport inhibitor response 1 (TIR1)-like protein  Transport inhibitor response 1 (TIR1)-like protein |
| miR396 | **Ta.33199 (1)**  **Ta.155884 (1)**  Ta.68649 (1)  **Ta.68429 (1)**  **Ta.36890 (1.5)**  Ta.68466 (2)  **CK209519 (2.5)** | Growth-regulating factor  Growth-regulating factor  Growth-regulating factor  Growth-regulating factor  Growth-regulating factor  Growth-regulating factor  Growth-regulating factor |
| miR408 | TC397865 (1)  TC427403 (2)  Ta.30891 (2)  **CA641607 (3)**  **TC391035 (1)**  **TC382360 (1)**  TC411642 (1.5) | Chemocyanin-like  Chemocyanin-like  Chemocyanin-like  Chemocyanin-like  Blue copper protein-like  Blue copper protein-like  Blue copper protein-like |
| miR444 | Ta.52424 (0)  Ta.47541 (0) | MIKC-type MADS-box transcription factor WM30  MIKC-type MADS-box transcription factor WM32A/B |
| miR827 | **Ta.88261 (2.5)**  Ta.48556 (3) | SPX domain-containing protein  Chaperone protein ClpD2 |
| miR894 | − | − |
| miR1878 | − | − |
| miR5048 | Ta.109260 (2)  Ta.48022 (2)  Ta.90063 (2)  Ta.10578 (2.5) | Cysteine-rich receptor-like protein kinase  Cysteine-rich receptor-like protein kinase  Cysteine-rich receptor-like protein kinase  Cysteine-rich receptor-like protein kinase |
| miR5062 | − | − |
| miR5064 | Ta.99041 (2)  Ta.105703 (2.5)  Ta.30223 (2.5) | Pentatricopeptide repeat (PPR) protein  Pentatricopeptide repeat (PPR) protein  Methionyl-tRNA synthetase |
| miR5071 | Ta.39113 (1.5)  Ta.75087 (1.5)  Ta.62345 (1.5)  Ta.105606 (1.5)  **Ta.92655 (2)**  **Ta.109875 (2)**  **Ta.111261 (3)** | Disease resistance protein RPM1-like  Disease resistance protein RPM1-like  Disease resistance protein RPP8-like  Disease resistance protein RPP8-like  Disease resistance protein RPP13-like  Disease resistance protein RPP8-like  Disease resistance protein RPP13-like |
| miR5139 | − | − |
| miR5175 | TC386363 (0.5)  TC394120 (1)  TC399088 (1)  TC426537 (2)  TC403274 (2)  TC397832 (2.5) | E3 ubiquitin-protein ligase HERC1  Dihydroflavonol-4-reductase  Omega-6 fatty acid desaturase  Glutaredoxin-C1  Transcription factor bHLH35-like  Exonuclease domain-containing protein 1 |
| miR6478 | − | − |
| miR9653a | TC370714 (2.5)  TC391108 (2.5)  TC400087 (2.5)  TC376279 (2.5) | Zinc finger protein 3-like  Zinc finger protein 3-like  Zinc finger protein 3-like  GTP-binding protein SAR1A |
| miR9653b | − | − |
| miR9655-5p | **TC431578 (1)** | Hypothetical protein |
| miR9658 | − | − |
| miR9659 | TC438538 (1.5)  TC382784 (3.5)  TC371921 (3.5) | Unknown  PIP1;5 protein  PIP1;5 protein |
| miR9662 | TC418804 (2)  TC453857 (2) | Mitochondrial transcription termination factor-like  Mitochondrial transcription termination factor-like |
| miR9666 | **TC389301 (2.5)**  **TC418574 (2.5)**  TC381864 (2.5) | DNA-directed RNA polymerase II  DNA-directed RNA polymerase II  Legumin-like protein |
| miR9669 | − | − |
| miR9670 | TC395665 (2)  TC439852 (2)  **TC453857 (2)**  TC419134 (3)  **TC421295 (3)** | Mitochondrial transcription termination factor-like  Mitochondrial transcription termination factor-like  Mitochondrial transcription termination factor-like  Mitochondrial transcription termination factor-like  Mitochondrial transcription termination factor-like |
| miR9672 | AJ603583 (0.5) | Brassinosteroid insensitive1-associated receptor kinase 1-like |
| miR9674 | Ta.102767 (2)  **Ta.216246 (2)**  Ta.112452 (2.5)  **Ta.66220 (3)**  **Ta.73044 (3)**  Ta.99041 (3)  Ta.100924 (3)  **Ta.113302 (3)** | PPR protein  PPR protein  PPR protein  PPR protein  PPR protein  PPR protein  PPR protein  PPR protein |
| miR9677 | Ta.95153 (1.5)  **Ta.47381 (2.5)**  **Ta.166036 (2.5)** | Hypothetical protein  Hypothetical protein  Hypothetical protein |
| miR9678 | Ta.73261 (2) | Unknown |
| miR9679 | Ta.34733 (2)  AJ603286 (2) | Unknown  Unknown |
| miR9772 | **Ta.144486 (2)**  **Ta.197955 (2)**  Ta.22291 (2.5)  **Ta.168249 (2.5)** | Predicted protein  Predicted protein  F-box protein  F-box protein |
| miR9773 | − | − |
| miR9774 | CK215494 (3) | Ribulose-1,5-bisphosphate carboxylase activase |
| miR9776 | Ta.25425 (3)  Ta.25863 (3) | 5'-adenylylsulfate reductase-like  F-box protein At4g17565-like |
| Tae-miR2008 | − | − |
| Tae-miR2009 | GH723869 (1)  Ta.108034 (1)  Ta.105298 (1)  Ta.121591 (1)  **Ta.78027 (1)**  **Ta.102789 (2)**  **Ta.109775 (2)**  **Ta.124901 (2)**  Ta.7078 (2.5)  Ta.72829 (2.5)  **TC402663 (4)**  **TC391850 (4)** | Disease resistance protein RPP13-like  Disease resistance protein RPP13-like  Disease resistance protein RPP13-like  Disease resistance protein RPP13-like  Disease resistance protein RPM1-like  Disease resistance protein RPM1-like  Disease resistance protein RPP8-like  Disease resistance protein RPP8-like  Disease resistance protein RPP8-like  Disease resistance protein RPP8-like  Unknown  Unknown |
| Ta-miR128 | Ta.41569 (3) | Unknown |
| Ta-miR154-5p | − | − |
| miR5048.2 | Ta.100067 (2)  **Ta.90063 (2)**  **Ta.40570 (2)**  **Ta.103967 (2.5)**  **Ta.115136 (2.5)**  Ta.48022 (2.5)  Ta.41454 (2.5)  Ta.10578 (3)  Ta.86927 (3)  Ta.109260 (3)  Ta.166907 (3) | Cysteine-rich receptor-like protein kinase  Cysteine-rich receptor-like protein kinase  Leucine-rich repeat receptor-like serine/threonine-protein kinase  Cysteine-rich receptor-like protein kinase  Cysteine-rich receptor-like protein kinase  Cysteine-rich receptor-like protein kinase  G-type lectin S-receptor-like serine/threonine-protein kinase  Cysteine-rich receptor-like protein kinase  Cysteine-rich receptor-like protein kinase  Cysteine-rich receptor-like protein kinase  Cysteine-rich receptor-like protein kinase |
| Ta-miRn1 | Ta.25217 (1.5)  Ta.57005 (2)  Ta.71280 (2.5)  **Ta.161175 (3)**  **Ta.55347 (3.5)**  **Ta.167568 (3.5)** | Serine/threonine-protein kinase RIO1  Regulator of Vps4 activity in the MVB pathway  Dehydrin  Coiled-coil domain containing protein (DUF2052)  Frigida-like protein  Disease resistance protein RPM1-like |
| Ta-miRn6 | Ta.78774 (2.5)  Ta.187583 (2.5) | Unknown  Unknown |
| Ta-miRn7 | TC420685 (0.5)  TC407053 (0.5)  TC388450 (2)  CJ883403 (2)  TC452182 (2)  TC381279 (2)  **TC433106 (2)**  **Ta.106901 (1)**  **Ta.76328 (2)** | 3-oxoacyl carrier protein synthase  NADPH-cytochrome P450 reductase  Aminolevulinic acid dehydratase  Cysteine synthase 2-like  Glyoxalase II  Metallo-beta-lactamase-like  Cell division protein ftsY homolog  Unknown  Cell division protein ftsY homolog |
| Ta-miRn9 | Ta.47541 (1.5)  Ta.52424 (3) | MIKC-type MADS-box transcription factor WM32A/B  MIKC-type MADS-box transcription factor WM30 |
| Ta-miRn13 | TC461985 (1)  TC436262 (2.5) | Mitogen-activated protein kinase (MAPK)  Cyclic nucleotide-gated ion channel 20 |
| Ta-miRn15 | TC397212 (0.5) | Eukaryotic peptide chain release factor subunit 1-3 |
| Ta-miRn17 | TC390615 (1)  TC403565 (1)  TC378689 (1)  TC446650 (1.5)  TC377378 (1.5)  TC440118 (2)  TC372628 (2)  **TC369686 (2)**  **Ta.76242 (2)**  **Ta.33781 (3)** | Homocysteine S-methyltransferase 3  Ubiquitin-like protein 5-like  Delta1-pyrroline-5-carboxylate synthetase  mtN19-like protein  mtN19-like protein  C2H2 zinc finger protein  Chlorophyll a/b-binding protein precursor  WD-40 repeat-containing protein  Mitochondrial-processing peptidase subunit alpha-like  Protein furry homolog-like protein |
| Ta-miRn19 | CV766835 (2) | Unknown |
| Ta-miRn21 | − | − |

# Targets in bold indicated that they were supported by degradome sequencing data.

^a^ Targets shown in red were confirmed by 5′ RACE.

Spaces separate parts of the table listing predicted targets of known miiRNAs (top) or novel miRNAs (bottom).
